# Duration–response association between occupational exposure and pancreatic cancer risk: meta-analysis

**DOI:** 10.1093/occmed/kqad050

**Published:** 2023-04-27

**Authors:** H Boonhat, A P Pratama, J-T Lin, R-T Lin

**Affiliations:** Graduate Institute of Public Health, College of Public Health, China Medical University, Taichung 406040, Taiwan; Graduate Institute of Public Health, College of Public Health, China Medical University, Taichung 406040, Taiwan; Division of Gastroenterology and Hepatology, Department of Internal Medicine, E-Da Hospital, Kaohsiung 824, Taiwan; Department of Occupational Safety and Health, College of Public Health, China Medical University, Taichung 406040, Taiwan

## Abstract

**Background:**

Evidence is lacking on the occupational exposure time window to chemical agents related to pancreatic cancer risk.

**Aims:**

This study performed meta-regression and meta-analysis to examine the dose–response association between occupational exposure duration to chemical agents and pancreatic cancer risk.

**Methods:**

We searched and reviewed studies on exposure duration and pancreatic cancer in five databases (Cochrane Library, EMBASE, PubMed, ScienceDirect and Web of Science) from inception to 16 May 2022. Exposure refers to the years a worker was exposed to any chemical agent, and outcome variables were pancreatic cancer incidence and mortality.

**Results:**

We identified 31 studies, including 288 389 participants. In the meta-regression, the positive dose–response association indicated pancreatic cancer risk increased slightly with every additional year of exposure duration (slope = 1.01; 95% confidence interval [CI] 1.00–1.02). Pancreatic cancer risk increased with an exposure duration of 1–10 (relative risk [RR] = 1.04; 95% CI 1.02–1.06), 11–20 (RR = 1.11; 95% CI 1.05–1.16), and 21–30 years (RR = 1.39; 95% CI 1.12–1.73).

**Conclusions:**

Pancreatic cancer risk increased as occupational exposure duration increased, with an exposure time window ranging from 1 to 30 years.

## Teaser

We observed a clear duration–response association between occupational exposure to chemical agents and pancreatic cancer risk. Our finding suggests a non-additional pancreatic cancer risk if the exposure time window decreased to less than 1 year, but the risk increased by 39% if the exposure time window increased to 21–30 years.

**Key learning points**

**What is already known about this subject:**

- Prolonged occupational exposures may pose serious health risks, especially for the development of cancer.
- There is limited evidence on the dose–response association between occupational exposure duration and pancreatic cancer risk.

**What this study adds:**

- Dose–response of occupational exposure duration and pancreatic cancer was observed.
- Our study suggests a non-additional risk of pancreatic cancer for occupational exposures <1 year, but an increase of 39% in risk for exposure durations of 21–30 years.
- Males exposed to Group 1 carcinogens had the highest pancreatic cancer risk.

**What impact this may have on practice or policy:**

- Future research should distinguish exposure duration from the latency period to identify the exposure time window of pancreatic cancer risk.
- Males exposes to Group 1 carcinogens for more than ten years should be monitored for symptoms and signs of pancreatic cancer.

## Introduction

Within the context of cancer, the term exposure duration represents the total period over which carcinogens might enter the human body [1], and it is an indicator used to regulate work–rest time [2]. Prolonged exposure to occupational risk factors may pose serious health risks, especially for the development of cancer [1]. The top five causes of cancer mortality globally in 2019 were lung, colorectal, stomach, breast and pancreatic cancers [3]. In previous meta-analyses, workers exposed to chemical agents for longer (e.g. 15 years) than shorter periods (e.g. 8 years) showed a higher risk of most cancers (including lung, colorectal, stomach and breast cancer) [4–7]. However, a pooled estimate of pancreatic cancer is lacking. Pancreatic cancer is typically found at an advanced stage and the survival rate is <10% [8], which is shorter than the other top four cancers [9]. Therefore, identifying occupational exposure duration to chemical agents and its association with pancreatic cancer may enhance our understanding of the exposure time window related to pancreatic cancer risk, and support the development of early identification indicators.

Although several studies have found a higher risk of pancreatic cancer with longer (10–20 or more years) than shorter exposure durations (1–10 years) [10,11], some studies provided contrary evidence. The effect of occupational exposure duration to chemical agents on pancreatic cancer risk may vary by exposure intensity (i.e. low and high), industry type [12] and chemical agents [13]. Across low and high exposure intensities, some studies reported a higher risk of pancreatic cancer in workers with a shorter exposure duration and high-intensity exposure to chemical agents than in those with prolonged but low-intensity exposure [14,15]. Regarding industry type, workers in the agricultural and automobile industries with shorter exposure duration had a higher risk of pancreatic cancer than those in the chemical industry with longer exposure duration [10,16]. Regarding chemical agents, workers exposed to pesticides for a shorter duration had a higher risk of pancreatic cancer than those exposed to ethylene oxide for a longer duration [17,18]. Exposure to chemical agents such as polycyclic aromatic hydrocarbons (PAHs), nickel and hydrocarbons may induce pancreatic cell trans-differentiation, enhance DNA changes and modulate oncogene expression, all of which are related to pancreatic carcinogenesis [19,20]. These occupational epidemiological studies indicated a paucity of information about the dose–response association between exposure duration and pancreatic cancer development.

Therefore, this study aimed to examine the dose–response association between occupational exposure duration to chemical agents and pancreatic cancer risk using meta-regression and meta-analysis. Specifically, we included all industry and chemical agent types to cover the widest possible range of working populations.

## Methods

We conducted a systematic search in five databases (Cochrane Library, EMBASE, PubMed, ScienceDirect and Web of Science) to identify epidemiological studies examining the association between exposure duration to chemical agents among workers, pancreatic cancer incidence and mortality until 16 May 2022. In this study, occupational exposure duration to chemical agents refers to the number of years that a worker was exposed to any chemical agent, and the outcomes were pancreatic cancer incidence and mortality.

The study question was formulated using the population, exposure, comparator (s), and outcomes (PECO) framework [21]: ‘Among workers, what is the effect of each yearly increase in occupational exposure duration to chemical agents on pancreatic cancer incidence and mortality?’ Details about the search terms are provided in Supplementary Material Section 1 (available as Supplementary data at *Occupational Medicine* Online). This meta-analysis was reported following the guidelines in the Preferred Reporting Items for Systematic Reviews and Meta-analyses (PRISMA; Supplementary Material Section 2, available as Supplementary data at *Occupational Medicine* Online) [22]. The search strategy was not language restricted but was limited to two article types: review and research articles. The eligibility criteria for inclusion and exclusion, the data transformation methods and data extraction are provided in Supplementary Material Sections 1 and 3 (available as Supplementary data at *Occupational Medicine* Online).

Following the methodology in the *Cochrane Handbook for Systematic Reviews of Interventions* [23], we used a natural log transformation of effect estimates to make the scale symmetric in the meta-regression and meta-analysis. We applied random-effects meta-regression to investigate the dose–response association between occupational exposure duration to chemical agents (continuous variable) and pancreatic cancer risk [23]. We further performed a random-effects meta-analysis to calculate the pooled estimates of relative risk (RR) and 95% confidence intervals (CIs) to identify the range of occupational exposure duration to chemical agents (categorical variable) associated with pancreatic cancer risk. The exposure time window refers to the exposure duration related to the outcome of interest [24], excluding the latency period.

We used employment years as a proxy for exposure duration to chemical agents. The range of occupational exposure duration to chemical agents was defined based on the original exposure duration of the selected studies. Specifically, most selected studies used 10 years as a cut-off. Therefore, we divided exposure duration range into 10-year intervals. However, some studies defined exposure duration as <1 year, and we classified them as <1 year to represent the lowest exposure. Therefore, exposure duration was classified as <1 year, 1–10 years, 11–20 years and 21–30 years. Some exposure durations in the selected studies did not meet our classification, so we calculated the mean and assigned them to one of the categories we established based on the mean exposure duration (e.g. in a study with an exposure duration ranging from 1 to 14 years, the mean is 7.5 years, so the exposure duration was classified as 1–10 years). Furthermore, some exposure durations in the selected studies did not account for the latency period for pancreatic cancer [25], so we assumed a latency of 10 years and subtracted this from exposure durations [16,17].

We further categorised occupational exposure duration to chemical agents into shorter (≤10 years) and longer durations (>10 years) to achieve approximately equal numbers of studies between the two periods. Subgroup analyses were conducted to compare the pooled effect estimates of occupational exposure duration to chemical agents (shorter versus longer) on pancreatic cancer risk by exposure intensity (high, low and not indicated), industry type (seven industries), chemical agent type (six agents), sex (female, male and combined), geographical areas (Asia, Europe and North America), risk of bias assessment (Tier 1 and Tier 2) and conflict of interest declaration (no, yes and not indicated). The risk of bias assessment information is provided in Supplementary Material Section 4 (available as Supplementary data at *Occupational Medicine* Online).

Given the relatively small differences in the numbers for pancreatic cancer incidence and mortality, we combined them as one target outcome, as in our previous meta-analysis [26]. The *I*-squared (*I*^2^) test was applied to explore effect heterogeneity across individual studies with the criteria of the degree of inconsistency as follows: *I*^2^ values of 0%–25%, 25%–50%, 50%–70% and >70% were defined as no, low, moderate and high heterogeneity, respectively [27]. The tau-squared (*τ*^2^) was used to identify between-study variations [23]. Funnel plots and Begg’s test were applied to examine publication bias [23].

A sensitivity analysis was performed to determine the consistency of the pooled RR between the main and alternative models of the meta-analysis. The main model was a meta-analysis of the association between occupational exposure duration to chemical agents and pancreatic cancer risk. The alternative model was a meta-analysis of the association between occupational exposure duration to chemical agents and pancreatic cancer risk by removing studies reporting the largest percentage of the total weight of pooled RR [28]. All statistical analyses were performed using Stata, version 16.1 (StataCorp, College Station, TX, USA). After the analyses, we evaluated the certainty of evidence and summarised in Supplementary Material Section 5 (available as Supplementary data at *Occupational Medicine* Online) [29]. This meta-analysis did not require ethical approval as it reviewed and summarised data in previously published literature.

## Results

**Figure 1.**
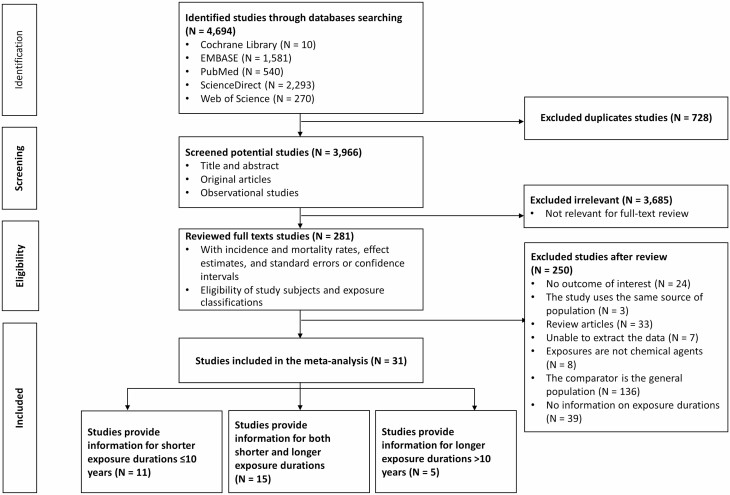
Flowchart of study selection. Abbreviation: *N*, number of studies.

A summary of the agreement rates during the screening and selection processes of the systematic review is provided in Supplementary Section 6 (available as Supplementary data at *Occupational Medicine* Online). The process of identifying studies in databases, screening for potential studies, evaluating full-text studies to assess eligibility, and including studies for meta-regression and meta-analysis is reported in Figure 1.

Among the 31 studies including 288 389 participants, 11 reported on shorter (≤10 years), 5 reported on longer (>10 years) and 15 reported on both occupational exposure durations to chemical agents. The characteristics of the 31 included studies are summarised in Supplementary Material Section 7 (available as Supplementary data at *Occupational Medicine* Online). Participants’ ages ranged from 23 to 85 years. All studies included in the analyses adjusted for age. Several (13/31 [42%]) adjusted for confounding variables including smoking, diabetes, obesity, socio-economic status and alcohol consumption.

**Figure 2.**
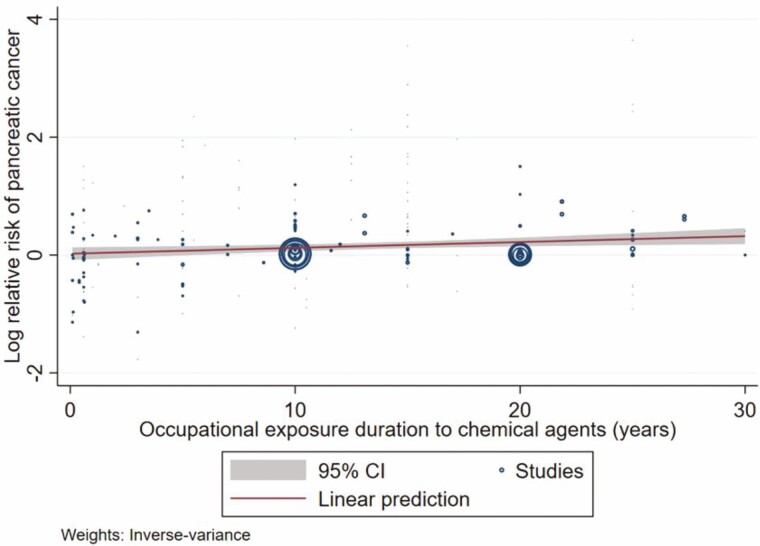
Meta-regression for the log relative risk of pancreatic cancer across different occupational exposure durations to chemical agents across the 31 included studies. Abbreviation: CI, confidence interval.

A meta-regression of 204 study groups across 31 studies was conducted to assess the dose–response association between occupational exposure duration to chemical agents and pancreatic cancer risk. The findings showed a significantly positive dose–response association between exposure duration and pancreatic cancer risk, with a 1% increase in pooled RR (slope = 1.01; 95% CI 1.00–1.02) per year (Figure 2). This finding suggests that longer occupational exposure to chemical agents is associated with an increased pancreatic cancer risk.

**Figure 3.**
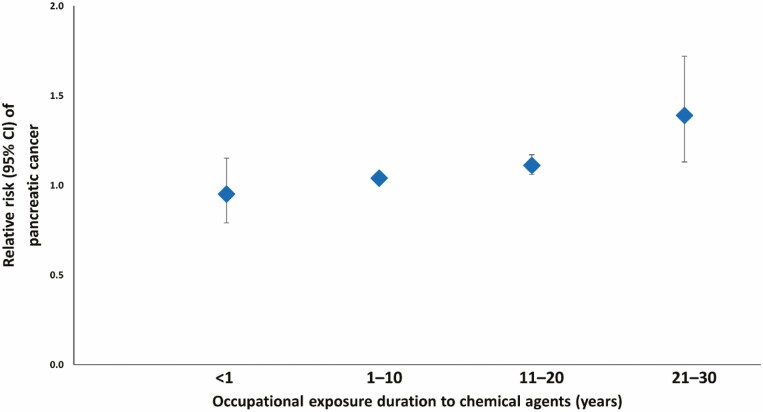
The pooled relative risk of pancreatic cancer by occupational exposure duration to chemical agents. Abbreviation: CI, confidence interval.

The meta-analysis revealed a significant association between occupational exposure duration to chemical agents and pancreatic cancer risk (pooled RR = 1.08; 95% CI 1.05–1.10; *I*^2^ = 51% and *τ*^2^ = 0.004). We observed a decreased risk of pancreatic cancer in workers exposed to chemical agents for <1 year, with a probability of null association (RR = 0.95; 95% CI 0.79–1.15; *I*^2^ = 29% and *τ*^2^ = 0.093; Figure 3). When considering prolonged exposure, we observed an increased risk of pancreatic cancer in workers exposed to chemical agents for 1–10 years (RR = 1.04; 95% CI 1.02–1.06; *I*^2^ = 31% and *τ*^2^ = 0.001), 11–20 years (RR = 1.11; 95% CI 1.06–1.17; *I*^2^ = 67% and *τ*^2^ = 0.008) and 21–30 years (RR = 1.39; 95% CI 1.13–1.72; *I*^2^ = 46% and *τ*^2^ = 0.082; Figure 3). The *I*^2^ for the exposure duration of 11–20 years indicated moderate heterogeneity among these studies, whereas the *I*^2^ for the remaining categories indicated low heterogeneity.

**Figure 4.**
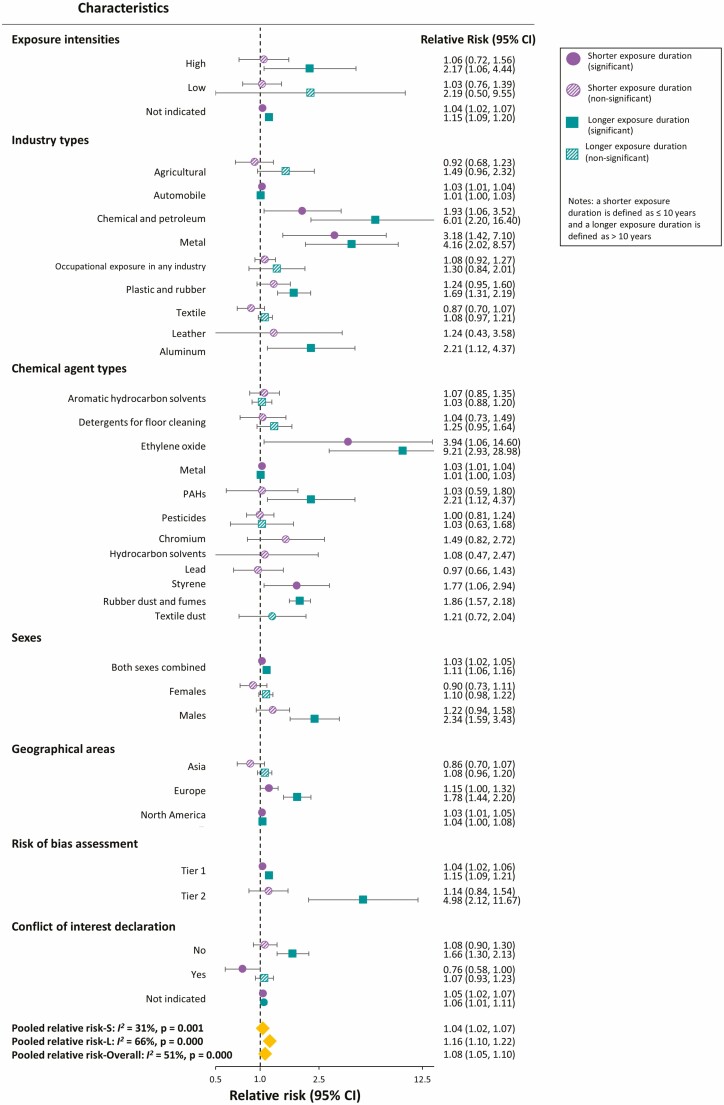
Subgroup analysis for the pooled relative risk of pancreatic cancer shorter and longer exposure durations by industry type, chemical agent type, geographical area, exposure assessment, intensity exposure level, risk of bias assessment and conflict of interest. Only one study was conducted in Oceania, so we excluded it from the geographical area subgroup analysis. Abbreviations: CI, confidence interval; *I*^2^, I-squared; L, longer exposure duration; PAHs, polycyclic aromatic hydrocarbons; S, shorter exposure duration.

Figure 4 shows the effect of occupational exposure to chemical agents on pancreatic cancer risk in subgroup analyses. Regarding exposure intensity, we found that the RRs of pancreatic cancer for both low and high exposure intensities were higher (and similar) for longer (RR = 2.19; 95% CI 0.50–9.55 and RR = 2.17; 95% CI 1.06–4.44, for low and high exposure intensities, respectively) than for shorter (RR = 1.03; 95% CI 0.76–1.39 and RR = 1.06; 95% CI 0.72–1.56, for low and high exposure intensities, respectively) exposure durations. Regarding industry type, the RRs of pancreatic cancer for most industries were also higher in longer than shorter exposure durations, especially for chemical, metal, and plastic and rubber industries. Regarding chemical agent type, we identified higher RRs of pancreatic cancer for longer than for shorter exposure durations for most types, particularly for ethylene oxide and PAHs.

The RRs of pancreatic cancer were also higher for longer than for shorter exposure durations in both male and female workers, all geographical areas, risk of bias assessment categories and regardless of whether the study reported conflicts of interest. However, studies with a conflict of interest had lower pooled RRs of pancreatic cancer than studies without a conflict of interest for both exposure durations.

**Figure 5.**
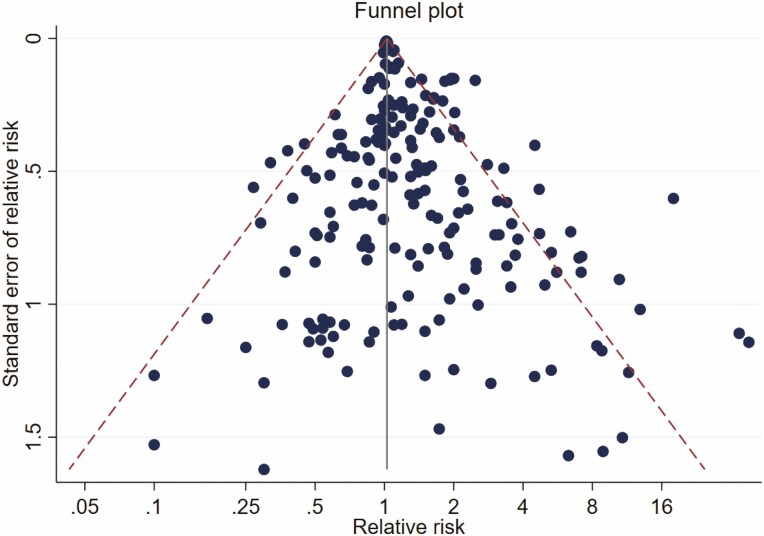
Funnel plot for relative risk of pancreatic cancer from the studies included in the meta-analysis of 204 study groups across 31 studies.

Overall, pooled RRs of pancreatic cancer for occupational exposure duration to chemical agents were 1.08 (95% CI 1.05–1.10; *I*^2^ = 51%) in the main meta-analysis model. One study [30] contributed the largest percentage to the total weight (78%; Supplementary Material Section 8, available as Supplementary data at *Occupational Medicine* Online). By excluding this study from the alternative meta-analysis model, pooled RRs of pancreatic cancer differed from the main model by approximately 15%, with an RR of 1.26 (95% CI 1.16–1.38; *I*^2^ = 50%). The *I*^2^ between the main and alternative models differed slightly, indicating moderate heterogeneity between the two models. The funnel plot for the meta-analysis showed symmetry for the effect estimates, and the Begg’s test suggested no publication bias (*P* = 0.768; Figure 5).

## Discussion

Our meta-regression showed that pancreatic cancer risk increased by a 1% per-year increment in occupational exposure duration to chemical agents, indicating a dose–response association. The certainty of evidence was moderate for an increased risk of pancreatic cancer. Subgroup analyses found pooled RRs of pancreatic cancer risk were higher for longer than for shorter exposure durations across most characteristics.

The major strengths of this study are: first, our inclusion of a large number (288 389) of participants from 31 published studies; and second, our limitation of healthy worker effect by comparing groups of workers, rather than the general population.

However, our meta-analysis has some limitations. We used employment years as a proxy for exposure duration. Cumulative exposure is a common summary measure used to quantify past exposure (i.e. the mean intensity and exposure duration in the past) [1,31].The environmental monitoring datasets used to estimate exposure intensity may sometimes be incomplete. One study described exposure duration as a stronger predictor of risk than exposure intensity [31]. Second, exposure age might impact pancreatic cancer development, and age was also a confounding factor [8]. However, all studies were adjusted for age, so this might not have greatly affected our study. Third, unadjusted confounding factors, such as smoking, alcohol, education and diabetes, may have influenced the findings, although 42% of the studies did adjust for these. Fourth, our sensitivity analysis showed a 15% difference between the pooled RRs for the main and alternative models, with one study contributing the most percentage to the total weight [30]. This may be because this cited study had a smaller standard error than the other studies, which in turn gave it more weight. At the same time, the *I*^2^ values did not diverge by model (main model: *I*^2^ = 51%; alternative model: *I*^2^ = 50%), suggesting that the cited study [30] might not have caused heterogeneity for the pooled RR of pancreatic cancer. Fifth, standard meta-regression and meta-analysis assume that the effect estimates of each exposure level are independent of each other. However, effect estimates of different exposure levels derived from the same study might be estimated by comparing with the same reference group (i.e. multiple study groups from a single study), indicating that the effect estimates could not be assumed to be independent [32]. If we assumed the effect estimates were independent, we might underestimate the variance of the duration–response slope [32]. A lower variance may weigh more on each study and lead to an overestimation of pooled effect estimates. So, we tried a different approach to investigate the duration–response association between occupational exposure and pancreatic cancer risk by combining the group effect of different exposure levels [33]. We observed a positive dose–response association with a 2% increase in the pooled RR per year (slope = 1.02; 95% CI 1.00–1.04; Supplementary Material Section 9, available as Supplementary data at *Occupational Medicine* Online). Since half [16] of the studies were excluded from the analysis, publication bias was present in the analysis. Therefore, we reported our results without publication bias.

Our results reflect two previous meta-analyses on the association between occupational exposure duration to chemical agents and four other types of cancer: which reported a significantly increased risk of lung and colorectal cancer in workers with > 20 years exposure to chemical agents [5,6]. Two meta-analyses reported no statistically significant dose-response association between occupational exposure duration to chemical agents and stomach cancer [4] or breast cancer [7]. These different results may be due to differences in exposure duration definitions. The meta-analyses by Guha et al [5] and Kwak et al [6] and ours considered only the number of years of occupational exposure to any chemical agent, whereas Fortunato and Rushton [4] and Marsh et al [7] summed both exposure duration and latency period. This finding suggests that having sufficient exposure duration and differentiating such duration from the latency period may be critical for accurately identifying the exposure interval for pancreatic cancer risk.

Low exposure intensity increased the risk of pancreatic cancer in workers with shorter and longer exposure duration by 1.03- and 2.19-fold, respectively. High exposure intensity increased the risk by 1.06- and 2.17-fold, respectively. So, our evidence demonstrates higher pancreatic cancer risk with longer than shorter occupational exposure durations, regardless of exposure intensity. Therefore, exposure duration could be an appropriate indicator when investigating past exposure to chemical agents and pancreatic cancer risk.

Chemical, metal, and plastic and rubber industries had higher RRs of pancreatic cancer risk for longer exposure durations. This is because the studies of these industries used an appropriate follow-up time (approximately 50, 30, and 40 years for the chemical, metal, and plastic and rubber industries, respectively) to obtain a relatively large number of pancreatic cancer cases for the dose-response association to be observed [10,34–36].

Industry-specific carcinogens may also increase pancreatic cancer risk. In the chemical industry, ethylene chlorohydrin (Group 2B, possibly carcinogenic to humans) is used to produce ethylene dichloride, which has been shown to increase the incidence of spleen, liver, pancreas and adrenal malignancies in animal studies [10,18,34]. Furthermore, accidental overexposure and spending more than 1 h per shift in the chlorohydrin room may increase pancreatic cancer risk [10,34]. In the metal industry, exposure to metal-working fluids used in metal machining and grinding may increase the risk of cancer, and potential carcinogens include PAHs in their straight and soluble forms, nitrosamines in soluble form and chlorinated oil [36,37].

Further, exposure to styrene in the reinforced plastics industry and to dust and fumes from rubber-making and vulcanisation processes in the rubber industry may increase cancer risk [38,39]. According to a rubber industry study, prolonged exposure to chemicals in vinyl and polyethylene processing, which involve potentially hazardous chemicals (e.g. formaldehyde, organic acids and solvents) may cause pancreatic cancer [35]. In this study [35], all related pancreatic cancer cases involved long-term workers on vinyl and polyethylene processing lines. Given that workers in the rubber industry may be exposed to multiple chemical agents for a prolonged duration, the synergistic effect of these multiple chemicals may increase pancreatic cancer risk. In our study, occupational exposure in any industry, and in agricultural, textile and automobile industries showed either non-significant or a difference in RRs of pancreatic cancer between shorter and longer exposure durations of less than 50%.

Ethylene oxide and PAHs had significantly higher RRs of pancreatic cancer for longer than shorter exposure durations in our analysis of six chemical agents. Other chemicals (detergents for floor cleaning, pesticides, aromatic hydrocarbons, and metals) showed either non-significant or a difference in RRs of pancreatic cancer between shorter and longer exposure durations of <50%. Ethylene oxide and PAHs are Group 1 carcinogens (carcinogenic to humans) [40], whereas other chemical agents are mostly Groups 2A (probably carcinogenic to humans) and 2B [41–43]. Our findings showed that prolonged exposure to Group 1 carcinogenic agents posed the highest risk to workers, whereas exposure to Group 2A or 2B agents showed a lower risk of pancreatic cancer, regardless of exposure duration. Moreover, we observed non-significant or a small difference in RRs owing to the mixture of chemicals used in detergents for floor cleaning, pesticides, aromatic hydrocarbons, and metal users. Our evidence implies that workers exposed to Group 1 carcinogens for longer should be considered at greater risk for pancreatic cancer.

Males had a higher risk of pancreatic cancer than females for both exposure durations. Research shows that lifestyle factors (e.g. smoking and alcohol) increased pancreatic cancer risk in men [44]. One study revealed that oestrogens in female rats inhibited early pancreatic carcinomatosis, while androgens in male rats may promote pancreatic cancer development [45]. Another explanation might be that female workers are more likely to be assigned to less hazardous areas than male workers [46]. We observed a positive association in three geographical areas, with the highest significant RRs of pancreatic cancer risk in Europe. This may be because most participants in European studies were male and most in Asian studies were female.

Regarding conflicts of interest, we observed that studies without conflicts of interest reported a positive RR, whereas studies with a conflict of interest reported a negative RR. Our meta-analysis revealed a positive dose–response association between occupational exposure duration to chemical agents and pancreatic cancer risk. Our study suggested no additional pancreatic cancer risk for exposure durations <1 year, but an additional risk of 39% for exposure durations of 21–30 years. Males exposed to Group 1 carcinogenic agents for more than 10 years should be monitored for symptoms and signs of pancreatic cancer.

## Supplementary Material

kqad050_suppl_Supplementary_Material

## References

[1] Checkoway H , Pearce N, Kriebel D. Exposure and Dose Modeling. Research Methods in Occupational Epidemiology. 1st edn. New York: Oxford University Press, 2004.

[2] ILO. Hours of Work (Industry) Convention, 1919 (No. 1): International Labour Organization; 1919. . https://www.ilo.org/dyn/normlex/en/f?p=NORMLEXPUB:12100:0::NO::P12100_ILO_CODE:C001 (23 June 2022, date last accessed).

[3] Kocarnik JM , Compton K, Dean FE et al. Cancer incidence, mortality, years of life lost, years lived with disability, and disability-adjusted life years for 29 cancer groups from 2010 to 2019: a systematic analysis for the Global Burden of Disease Study 2019. JAMA Oncol 2022;8:420–444. 34967848 10.1001/jamaoncol.2021.6987 PMC8719276

[4] Fortunato L , Rushton L. Stomach cancer and occupational exposure to asbestos: a meta-analysis of occupational cohort studies. Br J Cancer 2015;112:1805–1815. 25928706 10.1038/bjc.2014.599 PMC4647249

[5] Guha N , Merletti F, Steenland Nelson K, Altieri A, Cogliano V, Straif K. Lung cancer risk in painters: a meta-analysis. Environ Health Perspect 2010;118:303–312. 20064777 10.1289/ehp.0901402 PMC2854755

[6] Kwak K , Paek D, Zoh KE. Exposure to asbestos and the risk of colorectal cancer mortality: a systematic review and meta-analysis. Occup Environ Med 2019;76:861–871. 31594840 10.1136/oemed-2019-105735

[7] Marsh GM , Keeton KA, Riordan AS, Best EA, Benson SM. Ethylene oxide and risk of lympho-hematopoietic cancer and breast cancer: a systematic literature review and meta-analysis. Int Arch Occup Environ Health 2019;92:919–939. 31111206 10.1007/s00420-019-01438-z

[8] Hu JX , Zhao CF, Chen WB et al. Pancreatic cancer: a review of epidemiology, trend, and risk factors. World J Gastroenterol 2021;27:4298–4321. 34366606 10.3748/wjg.v27.i27.4298 PMC8316912

[9] Allemani C , Weir HK, Carreira H et al. Global surveillance of cancer survival 1995–2009: analysis of individual data for 25,676,887 patients from 279 population-based registries in 67 countries (CONCORD-2). Lancet 2015;385:977–1010. 25467588 10.1016/S0140-6736(14)62038-9 PMC4588097

[10] Benson LO , Teta MJ. Mortality due to pancreatic and lymphopoietic cancers in chlorohydrin production workers. Br J Ind Med 1993;50:710–716. 8398857 10.1136/oem.50.8.710 PMC1012174

[11] Marsh GM , Youk AO, Buchanich JM, Kant IJ, Swaen G. Mortality patterns among workers exposed to acrylamide: updated follow up. J Occup Environ Med 2007;49:82–95. 17215717 10.1097/JOM.0b013e31802db536

[12] Lynge E , Andersen A, Rylander L et al. Cancer in persons working in dry cleaning in the Nordic countries. Environ Health Perspect 2006;114:213–219. 16451857 10.1289/ehp.8425 PMC1367834

[13] Reul NK , Li W, Gallagher LG et al. Risk of pancreatic cancer in female textile workers in Shanghai, China, exposed to metals, solvents, chemicals, and endotoxin: follow-up to a nested case-cohort study. J Occup Environ Med 2016;58:195–199. 26849264 10.1097/JOM.0000000000000596 PMC4870312

[14] Alguacil J , Kauppinen T, Porta M et al. Risk of pancreatic cancer and occupational exposures in Spain: PANKRAS II Study Group. Ann Occup Hyg 2000;44:391–403. 10930502

[15] Kolstad HA , Juel K, Olsen J, Lynge E. Exposure to styrene and chronic health effects: mortality and incidence of solid cancers in the Danish reinforced plastics industry. Occup Environ Med 1995;52:320–327. 7795754 10.1136/oem.52.5.320 PMC1128224

[16] Alguacil J , Porta M, Benavides FG et al. Occupation and pancreatic cancer in Spain: a case-control study based on job titles. Int J Epidemio 2000;29:1004–1013. 10.1093/ije/29.6.1004 11101541

[17] Beard J , Sladden T, Morgan G, Berry G, Brooks L, McMichael A. Health impacts of pesticide exposure in a cohort of outdoor workers. Environ Health Perspect 2003;111:724–730. 12727601 10.1289/ehp.5885 PMC1241482

[18] Teta MJ , Benson LO, Vitale JN. Mortality study of ethylene oxide workers in chemical manufacturing: a 10 year update. Br J Ind Med 1993;50:704–709. 8398856 10.1136/oem.50.8.704 PMC1012173

[19] Mehlman MA. Dangerous and cancer-causing properties of products and chemicals in the oil refining and petrochemical industry: VIII. Health effects of motor fuels: carcinogenicity of gasoline—scientific update. Environ Res 1992;59:238–249. 1425514 10.1016/s0013-9351(05)80243-9

[20] Mehlman MA , Legator MS. Dangerous and cancer-causing properties of products and chemicals in the oil refining and petrochemical industry—part II: carcinogenicity, mutagenicity, and developmental toxicity of 1,3-butadiene. Toxicol Ind Health 1991;7:207–220. 1949058 10.1177/074823379100700306

[21] Morgan RL , Whaley P, Thayer KA, Schünemann HJ. Identifying the PECO: a framework for formulating good questions to explore the association of environmental and other exposures with health outcomes. Environ Int 2018;121:1027–1031. 30166065 10.1016/j.envint.2018.07.015 PMC6908441

[22] Page MJ , McKenzie JE, Bossuyt PM et al. The PRISMA 2020 statement: an updated guideline for reporting systematic reviews. Br Med J 2021;372:n71. 33782057 10.1136/bmj.n71 PMC8005924

[23] Higgins JPT , Chandler J, Cumpston M, Li T, Page M, Welch V. Cochrane Handbook for Systematic Reviews of Interventions Version 6.1 (Updated September 2020). Chichester (UK): John Wiley & Sons. 2020. www.training.cochrane.org/handbook (22 July 2022, date last accessed).

[24] Lee TA , Pickard AS. Exposure definition and measurement. In: Velentgas P, Dreyer NA, Nourjah P, et al., eds. Developing a Protocol for Observational Comparative Effectiveness Research: A User’s Guide. Rockville: Agency for Healthcare Research and Quality (US), 2013. https://www.ncbi.nlm.nih.gov/books/NBK126191/ (22 July 2022, date last accessed). 23469377

[25] van Barneveld TA , Sasco AJ, van Leeuwen FE. A cohort study of cancer mortality among Biology Research Laboratory workers in the Netherlands. Cancer Causes Control 2004;15:55–66. 14970735 10.1023/B:CACO.0000016607.70457.47

[26] Boonhat H , Lin RT, Lin JT. Association between residential exposure to petrochemical industrial complexes and pancreatic cancer: a systematic review and meta-analysis. Int J Environ Health Res 2023;33:116–127. 34930088 10.1080/09603123.2021.2007226

[27] Higgins JPT. Commentary: heterogeneity in meta-analysis should be expected and appropriately quantified. Int J Epidemiol 2008;37:1158–1160. 18832388 10.1093/ije/dyn204

[28] Van Maele-Fabry G , Hoet P, Vilain F, Lison D. Occupational exposure to pesticides and Parkinson’s disease: a systematic review and meta-analysis of cohort studies. Environ Int 2012;46:30–43. 22698719 10.1016/j.envint.2012.05.004

[29] Schünemann HJ , Vist GE, Glasziou P, Akl EA, Skoetz N, Guyatt GH. Chapter 14: completing ‘summary of findings’ tables and grading the certainty of the evidence. In: Higgins JPT, Thomas J, Chandler J, et al., eds. Cochrane Handbook for Systematic Reviews of Interventions Version 6.1 (Updated September 2020). Chichester: John Wiley & Son, 2020. www.training.cochrane.org/handbook (18 September 2022, date last accessed).

[30] Bardin JA , Eisen EA, Tolbert PE et al. Mortality studies of machining fluid exposure in the automobile industry. V: a case-control study of pancreatic cancer. Am J Ind Med 1997;32:240–247. 9219653 10.1002/(sici)1097-0274(199709)32:3<240::aid-ajim9>3.0.co;2-0

[31] de Vocht F , Burstyn I, Sanguanchaiyakrit N. Rethinking cumulative exposure in epidemiology, again. J Expo Sci Environ Epidemiol 2015;25:467–473. 25138292 10.1038/jes.2014.58

[32] Greenland S , Longnecker MP. Methods for trend estimation from summarized dose-response data, with applications to meta-analysis. Am J Epidemiol 1992;135:1301–1309. 1626547 10.1093/oxfordjournals.aje.a116237

[33] Orsini N , Li R, Wolk A, Khudyakov P, Spiegelman D. Meta-analysis for linear and nonlinear dose-response relations: examples, an evaluation of approximations, and software. Am J Epidemiol 2012;175:66–73. 22135359 10.1093/aje/kwr265 PMC3244608

[34] Greenberg HL , Ott MG, Shore RE. Men assigned to ethylene oxide production or other ethylene oxide related chemical manufacturing: a mortality study. Br J Ind Med 1990;47:221–230. 2337530 10.1136/oem.47.4.221 PMC1035141

[35] Selenskas S , Teta MJ, Vitale JN. Pancreatic cancer among workers processing synthetic resins. Am J Ind Med 1995;28:385–398. 7485192 10.1002/ajim.4700280308

[36] Silverstein M , Park R, Marmor M, Maizlish N, Mirer F. Mortality among bearing plant workers exposed to metalworking fluids and abrasives. J Occup Med 1988;30:706–714. 3183787

[37] IARC. ARC Monographs on the Evaluation of Carcinogenic Risks to Humans, Volume 77, Some Industrial Chemicals. Lyon: International Agency for Research on Cancer, 2000. https://www.ncbi.nlm.nih.gov/books/NBK464353/9 September 2022, date last accessed).

[38] IARC. Chemical Agents and Related Occupations: Occupational Exposures in the Rubber-Manufacturing Industry. Lyon: International Agency for Research on Cancer, 2012. https://www.ncbi.nlm.nih.gov/books/NBK304412/ (9 September 2022, date last accessed).

[39] IARC. Styrene, Styrene-7,8-Oxide, and Quinoline. Lyon: International Agency for Research on Cancer, 2019. https://www.ncbi.nlm.nih.gov/books/NBK551034/ (9 September 2022, date last accessed). 31967769

[40] IARC. Chemical Agents and Related Occupations. Lyon: International Agency for Research on Cancer, 2012 https://www.ncbi.nlm.nih.gov/books/NBK304416/ (9 September 2022, date last accessed).

[41] IARC. Beryllium, Cadmium, Mercury, and Exposures in the Glass Manufacturing Industry. Lyon: International Agency for Research on Cancer, 1993. https://www.ncbi.nlm.nih.gov/books/NBK499780/ (9 September 2022, date last accessed).

[42] IARC. Dry Cleaning, Some Chlorinated Solvents and Other Industrial Chemicals. Lyon: International Agency for Research on Cancer, 1995. https://www.ncbi.nlm.nih.gov/books/NBK464358/ (9 September 2022, date last accessed).

[43] IARC. Some Organophosphate Insecticides and Herbicides. Lyon: International Agency for Research on Cancer; 2017. https://www.ncbi.nlm.nih.gov/books/NBK436757/ (9 September 2022, date last accessed). 31829533

[44] Rawla P , Sunkara T, Gaduputi V. Epidemiology of pancreatic cancer: global trends, etiology and risk factors. World J Oncol 2019;10:10–27. 30834048 10.14740/wjon1166 PMC6396775

[45] Wang M , Gorelick F, Bhargava A. Sex differences in the exocrine pancreas and associated diseases. Cell Mol Gastroenterol Hepatol 2021;12:427–441. 33895424 10.1016/j.jcmgh.2021.04.005 PMC8255941

[46] Santibañez M , Vioque J, Alguacil J et al. Occupational exposures and risk of pancreatic cancer. Eur J Epidemiol 2010;25:721–730. 20640489 10.1007/s10654-010-9490-0

