# Supplementary material for: Duration–response association between occupational exposure and pancreatic cancer risk: meta-analysis
Source: Occup Med (Lond). 2023 Apr 27;73(4):211–8. doi: 10.1093/occmed/kqad050 (PMC10195200; doi:10.1093/occmed/kqad050)
Supplement: kqad050_suppl_Supplementary_Material [file kqad050_suppl_supplementary_material.doc]

**Supplementary Materials**

[**Section 1: Search strategy** 2](#__RefHeading___Toc127115594)

[**Section 2: The Preferred Reporting Items for Systematic Reviews and Meta-Analyses (PRISMA)** 4](#__RefHeading___Toc127115595)

[**Section 3: Data transformation** 12](#__RefHeading___Toc127115596)

[**Section 4: Risk of bias assessment** 13](#__RefHeading___Toc127115597)

[**Section 5: Certainty of evidence** 48](#__RefHeading___Toc127115598)

[**Section 6: The flowchart for agreement during screening and selection of systematic review** 50](#__RefHeading___Toc127115599)

[**Section 7: The characteristics of studies included in the meta-regression and meta-analysis** 51](#__RefHeading___Toc127115600)

[**Section 8: The percentage of total weight across the 31 included studies** 65](#__RefHeading___Toc127115601)

[**Section 9: Duration-response association between occupational exposure to chemical agent and pancreatic cancer risk by weighted random-effect dose response model** 66](#__RefHeading___Toc127115602)

# **Section 1: Search strategy**

**Table S1.** Electronic search strategy.

| **Databases** | **Search terms** |
| --- | --- |
| **Cochrane Library**  **(Field: All text)** | (“Occupational exposure” OR “Occupational risk factors” OR Occupation OR Worker) AND (“Pancreatic Neoplasm” OR “Pancreas Neoplasm*” OR “Pancreas Cancer*” OR “Pancreatic Cancer*” OR “Cancer of Pancreas” OR “Cancer of the Pancreas”) |
| **EMBASE** | (Occupational exposure OR Occupational risk factors OR Occupation OR Worker) AND (Pancreatic Neoplasm OR Pancreas Neoplasm OR Pancreas Cancer OR Pancreatic Cancer OR Cancer of Pancreas OR Cancer of the Pancreas) |
| **PubMed** | (“Occupational exposure” OR “Occupational risk factors” OR Occupation OR Worker) AND (“Pancreatic Neoplasm” OR “Pancreas Neoplasm*” OR “Pancreas Cancer*” OR “Pancreatic Cancer*” OR “Cancer of Pancreas” OR “Cancer of the Pancreas”) |
| **ScienceDirect**  **(Article types: Review articles and research articles)**  **#1** | (“Occupational exposure” OR “Occupational risk factors” OR Occupation OR Worker) AND (“Pancreatic Neoplasm” OR “Pancreas Neoplasm” OR “Pancreas Cancer” OR “Pancreatic Cancer”) |
| **#2** | (“Occupational exposure” OR “Occupational risk factors” OR Occupation OR Worker) AND (“Cancer of Pancreas” OR “Cancer of the Pancreas”) |
| **Web of Science** | ((“Occupational exposure” OR “Occupational risk factors” OR Occupation OR Worker) AND (“Pancreatic Neoplasm” OR “Pancreas Neoplasm*” OR “Pancreas Cancer*” OR “Pancreatic Cancer*” OR “Cancer of Pancreas” OR “Cancer of the Pancreas”)) |

The eligibility criteria for inclusion in this meta-analysis were as follows: 1) studies that defined the exposure group as workers who had worked in any industry or occupation and were exposed to any chemical agents; 2) that defined the comparator group as workers who had worked in different industries or occupations in comparison to the exposure group, or workers who had not been exposed to chemical agents; 3) that classified an outcome as pancreatic cancer incidence or mortality based on the International Classification of Diseases (ICD) in any of its versions, histological confirmation, death certificate, hospital records, or cancer registry.

The related exclusion criteria were as follows: 1) studies with no outcome variable of pancreatic cancer incidence or mortality; 2) that used the same study population (in the case of the same study population, we selected studies with a longer study follow-up and a larger number of subjects); 3) conference papers; 4) studies that either did not report effect measures or provided insufficient information to calculate these measures; 5) that analyzed exposures to chemical agents not related to any industry (e.g., ionizing radiation); 6) that included a comparator group comprising people from the general population; 7) that did not adjust for confounding factors; 8) that did not provide information on exposure duration. Study type was not restricted.

Two researchers (HB and AP) independently conducted the literature search, screened titles and abstracts, and assessed study eligibility based on the inclusion and exclusion criteria for the meta-analysis. A reference manager software (Endnote, version X9) was utilized to exclude duplicate studies. To avoid missing any related studies, we searched for research related to occupational exposure and pancreatic cancer in the references lists of the review articles that were included in the full-text review process. Disagreements between the two researchers on whether to include studies in the review and meta-analysis were resolved by discussion. If both researchers could not reach consensus, a senior researcher (RTL) made the final decision. The data transformation methods and data extraction are provided in Supplementary Material Section 3.

# **Section 2: The Preferred Reporting Items for Systematic Reviews and Meta-Analyses (PRISMA)**

**Table S2.** PRISMA 2020 checklist for the transparent reporting of systematic reviews and meta-analyses.

| **Topic** | **No.** | **Item** | **Location where item is reported** |
| --- | --- | --- | --- |
| **TITLE** |  |  |  |
| **Title** | 1 | Identify the report as a systematic review. | Page 1, line numbers 1–2 |
| **ABSTRACT** |  |  |  |
| **Abstract** | 2 | See the PRISMA 2020 for Abstracts checklist |  |
| **INTRODUCTION** |  |  |  |
| **Rationale** | 3 | Describe the rationale for the review in the context of existing knowledge. | Pages 4–5, line numbers 47–79 |
| **Objectives** | 4 | Provide an explicit statement of the objective(s) or question(s) the review addresses. | Page 5, line numbers 81–86 |
| **METHODS** |  |  |  |
| **Eligibility criteria** | 5 | Specify the inclusion and exclusion criteria for the review and how studies were grouped for the syntheses. | Supplementary material Section 1 |
| **Information sources** | 6 | Specify all databases, registers, websites, organisations, reference lists and other sources searched or consulted to identify studies. Specify the date when each source was last searched or consulted. | Page 5, line numbers 89–90 |
| **Search strategy** | 7 | Present the full search strategies for all databases, registers and websites, including any filters and limits used. | Page 5, line numbers 89–90 and Supplementary material Section 1 |
| **Selection process** | 8 | Specify the methods used to decide whether a study met the inclusion criteria of the review, including how many reviewers screened each record and each report retrieved, whether they worked independently, and if applicable, details of automation tools used in the process. | Supplementary material Section 1 |
| **Data collection process** | 9 | Specify the methods used to collect data from reports, including how many reviewers collected data from each report, whether they worked independently, any processes for obtaining or confirming data from study investigators, and if applicable, details of automation tools used in the process. | Supplementary material Section 3 |
| **Data items** | 10a | List and define all outcomes for which data were sought. Specify whether all results that were compatible with each outcome domain in each study were sought (e.g. for all measures, time points, analyses), and if not, the methods used to decide which results to collect. | Supplementary material Section 3 |
|  | 10b | List and define all other variables for which data were sought (e.g. participant and intervention characteristics, funding sources). Describe any assumptions made about any missing or unclear information. | Supplementary material Section 3 |
| **Study risk of bias assessment** | 11 | Specify the methods used to assess risk of bias in the included studies, including details of the tool(s) used, how many reviewers assessed each study and whether they worked independently, and if applicable, details of automation tools used in the process. | Page 8, line numbers 140–141 and Supplementary material Section 4 |
| **Effect measures** | 12 | Specify for each outcome the effect measure(s) (e.g. risk ratio, mean difference) used in the synthesis or presentation of results. | Page 6, line numbers 110–113 |
| **Synthesis methods** | 13a | Describe the processes used to decide which studies were eligible for each synthesis (e.g. tabulating the study intervention characteristics and comparing against the planned groups for each synthesis (item 5)). | Page 7, line numbers 117–131 |
|  | 13b | Describe any methods required to prepare the data for presentation or synthesis, such as handling of missing summary statistics, or data conversions. | Supplementary material Section 3 |
|  | 13c | Describe any methods used to tabulate or visually display results of individual studies and syntheses. | Page 6, line numbers 106–113 |
|  | 13d | Describe any methods used to synthesize results and provide a rationale for the choice(s). If meta-analysis was performed, describe the model(s), method(s) to identify the presence and extent of statistical heterogeneity, and software package(s) used. | Page 8, line numbers 143–149 and 156–157 |
|  | 13e | Describe any methods used to explore possible causes of heterogeneity among study results (e.g. subgroup analysis, meta-regression). | Pages 7–8, line numbers 133–141 |
|  | 13f | Describe any sensitivity analyses conducted to assess robustness of the synthesized results. | Page 8, line numbers 151–156 |
| **Reporting bias assessment** | 14 | Describe any methods used to assess risk of bias due to missing results in a synthesis (arising from reporting biases). | Page 8, line numbers 140–141 and Supplementary material Section 4 |
| **Certainty assessment** | 15 | Describe any methods used to assess certainty (or confidence) in the body of evidence for an outcome. | Page 8, line numbers 157–158 and Supplementary material Section 5 |
| **RESULTS** |  |  |  |
| **Study selection** | 16a | Describe the results of the search and selection process, from the number of records identified in the search to the number of studies included in the review, ideally using a flow diagram. | Page 9, line numbers 162–165 |
|  | 16b | Cite studies that might appear to meet the inclusion criteria, but which were excluded, and explain why they were excluded. | Not reported |
| **Study characteristics** | 17 | Cite each included study and present its characteristics. | Page 9, line numbers 167–173 and Supplementary material section 7 |
| **Risk of bias in studies** | 18 | Present assessments of risk of bias for each included study. | Supplementary material section 4 |
| **Results of individual studies** | 19 | For all outcomes, present, for each study: (a) summary statistics for each group (where appropriate) and (b) an effect estimates and its precision (e.g. confidence/credible interval), ideally using structured tables or plots. | Supplementary material section 7 |
| **Results of syntheses** | 20a | For each synthesis, briefly summarise the characteristics and risk of bias among contributing studies. | Supplementary material section 4 |
|  | 20b | Present results of all statistical syntheses conducted. If meta-analysis was done, present for each the summary estimate and its precision (e.g. confidence/credible interval) and measures of statistical heterogeneity. If comparing groups, describe the direction of the effect. | Pages 9–11, line numbers 175–221 |
|  | 20c | Present results of all investigations of possible causes of heterogeneity among study results. | Pages 9–11, line numbers 175–221 |
|  | 20d | Present results of all sensitivity analyses conducted to assess the robustness of the synthesized results. | Page 11, line numbers 213–221 |
| **Reporting biases** | 21 | Present assessments of risk of bias due to missing results (arising from reporting biases) for each synthesis assessed. | Supplementary material Section 4 |
| **Certainty of evidence** | 22 | Present assessments of certainty (or confidence) in the body of evidence for each outcome assessed. | Supplementary material Section 5 |
| **DISCUSSION** |  |  |  |
| **Discussion** | 23a | Provide a general interpretation of the results in the context of other evidence. | Page 12, line numbers 223–228 |
|  | 23b | Discuss any limitations of the evidence included in the review. | Pages 12–13, line numbers 234–257 |
|  | 23c | Discuss any limitations of the review processes used. | Not reported |
|  | 23d | Discuss implications of the results for practice, policy, and future research. | Page 17, line numbers 342–348 |
| **OTHER INFORMATION** |  |  |  |
| **Registration and protocol** | 24a | Provide registration information for the review, including register name and registration number, or state that the review was not registered. | The review was not registered |
|  | 24b | Indicate where the review protocol can be accessed, or state that a protocol was not prepared. | A protocol was not prepared |
|  | 24c | Describe and explain any amendments to information provided at registration or in the protocol. | Not reported |
| **Support** | 25 | Describe sources of financial or non-financial support for the review, and the role of the funders or sponsors in the review. | Page 19, line numbers 370–372 |
| **Competing interests** | 26 | Declare any competing interests of review authors. | Pages 19, line numbers 381–383 |
| **Availability of data, code and other materials** | 27 | Report which of the following are publicly available and where they can be found: template data collection forms; data extracted from included studies; data used for all analyses; analytic code; any other materials used in the review. | Not reported |

*From:* Page MJ, McKenzie JE, Bossuyt PM, Boutron I, Hoffmann TC, Mulrow CD, et al. The PRISMA 2020 statement: an updated guideline for reporting systematic reviews. MetaArXiv. 2020, September 14. DOI: 10.31222/osf.io/v7gm2. For more information, visit: [www.prisma-statement.org](../../../../C:/Users/Angela/AppData/Local/Microsoft/Windows/INetCache/Content.Outlook/5J0JOEAY/www.prisma-statement.org)

**Table S2.** PRISMA 2020 checklist for the transparent reporting of systematic reviews and meta-analyses (for abtract).

| **Topic** | **No.** | **Item** | **Reported?** |
| --- | --- | --- | --- |
| **TITLE** |  |  |  |
| **Title** | 1 | Identify the report as a systematic review. | Yes |
| **BACKGROUND** |  |  |  |
| **Objectives** | 2 | Provide an explicit statement of the main objective(s) or question(s) the review addresses. | Yes |
| **METHODS** |  |  |  |
| **Eligibility criteria** | 3 | Specify the inclusion and exclusion criteria for the review. | No |
| **Information sources** | 4 | Specify the information sources (e.g. databases, registers) used to identify studies and the date when each was last searched. | Yes |
| **Risk of bias** | 5 | Specify the methods used to assess risk of bias in the included studies. | No |
| **Synthesis of results** | 6 | Specify the methods used to present and synthesize results. | Yes |
| **RESULTS** |  |  |  |
| **Included studies** | 7 | Give the total number of included studies and participants and summarise relevant characteristics of studies. | Yes |
| **Synthesis of results** | 8 | Present results for main outcomes, preferably indicating the number of included studies and participants for each. If meta-analysis was done, report the summary estimate and confidence/credible interval. If comparing groups, indicate the direction of the effect (i.e. which group is favoured). | Yes |
| **DISCUSSION** |  |  |  |
| **Limitations of evidence** | 9 | Provide a brief summary of the limitations of the evidence included in the review (e.g. study risk of bias, inconsistency and imprecision). | No |
| **Interpretation** | 10 | Provide a general interpretation of the results and important implications. | Yes |
| **OTHER** |  |  |  |
| **Funding** | 11 | Specify the primary source of funding for the review. | No |
| **Registration** | 12 | Provide the register name and registration number. | No |

*From:* Page MJ, McKenzie JE, Bossuyt PM, Boutron I, Hoffmann TC, Mulrow CD, et al. The PRISMA 2020 statement: an updated guideline for reporting systematic reviews. MetaArXiv. 2020, September 14. DOI: 10.31222/osf.io/v7gm2. For more information, visit: [www.prisma-statement.org](../../../../C:%5CUsers%5Clenovo%5CDownloads%5Cwww.prisma-statement.org)

# **Section 3: Data transformation**

**Table S3.** The formula for standard error calculation.

Information of eligible studies was extracted using a Microsoft Excel spreadsheet, including the following: 1) author’s name, 2) publication year, 3) country, 4) exposure intensity, 5) industry type/occupations, 6) related-chemical agents, 7) participant enrollment, 8) study types, 9) effect measure types, 10) number of effect measures, 11) confounders, 12) age of participants, 13) sex, 14) number of participants, 15) exposure assessment, 16) employment years, 17) outcome assessment, and 18) declaration of conflict of interest.

| To perform a meta-regression and a meta-analysis, we used the number of effect measures to pool the effect estimates. However, the selected studies used a diversity of effect measures, including the relative risk (RR), the odds ratio (OR), and the hazard ratio (HR). So, we pooled and reported the effect estimates into RRs according to the following two reasons. First, the OR were considered to be equivalent to the RR when pancreatic cancer is a rare disease (1). Second, the HR were considered to be approximate to the RR when follow-up is short and event rates are small (2). We calculated the standard error (SE) of the natural log of RR as the following formula:   | SE (lnRR) = 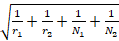 | (1) | | --- | --- |   where 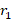, 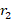, 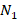, and 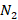 are the number of workers in the industries/occupations among workers who were exposed to the chemical agent and developed pancreatic cancer, the number of workers in the industries/occupations among workers who were not exposed to the chemical agent and developed pancreatic cancer, the total number of workers in the industries/occupations among workers who were exposed to the chemical agent, and the total number of workers in the industries/occupations among workers who were not exposed to the chemical agents, respectively. |
| --- | --- | --- |

# **Section 4: Risk of bias assessment**

We assessed the risk of bias for individual studies according to the Office of Health Assessment and Translation (OHAT) (3)to categorize each individual study into tiers 1–3 for risk of bias to support our final conclusion. The OHAT risk of bias includes seven domains, such as confounding, detection, and selection biases. The risk of bias for each study was assessed as definitely low, probably low, probably high, and definitely high. When there was insufficient information to ascertain the individual domains, the studies were rated as having a probably high risk of bias. The two researchers (HB and AP) independently performed the risk of bias assessment. In cases of disagreement, the senior researcher (RTL) joined the discussion and resolved the disagreement.

The OHAT’s risk of bias tier considers some key elements as follows:

- Does the study design or analysis account for important confounding variables?
- Can we be confident in the exposure characterization?
- Can we be confident in the outcome assessment?

Tier are classified as follows:

**Tier 1:** A study must be rated as “definitely low” or “probably low” risk of bias for key elements AND have most other applicable items answered as “definitely low” or “probably low” risk of bias.

**Tier 2:** A study meets neither of the criteria for tiers.

**Tier 3:** A study must be rated as “definitely high” or “probably high” risk of bias for key elements AND have most other applicable items answered as “definitely high” or “probably high” risk of bias.

There are criteria to integrate the risk of bias from each study, providing relevant information for the health outcomes of interest.

| **Rating** | **Definition** |
| --- | --- |
| “Not likely” | Most of the information is from Tier 1 studies (low risk of bias for all key domains).  Plausible bias is unlikely to seriously alter the results. |
| “Serious” | Most of the information is from Tiers 1 and 2 studies.  Plausible bias that raises some doubt about the results. |
| “Very serious” | The proportion of information from Tier 3 studies at high risk of bias for all key domains is sufficient to affect the interpretation of results.  Plausible bias that seriously weakens confidence in the results. |

We summarized the risk-of-bias assessment in Table S4, and the rationale for each evaluation is shown in Tables S4A–S4Ae. We used the OHAT risk-of-bias tool to categorize each study by the domain of bias. Confounding and detection biases were considered as the most important domains for this risk-of-bias tool. Other biases (selection, attrition/exclusion, selective reporting, and other biases) did not have a significant influence on the overall risk-of-bias.

Confounding bias was rated as “probably high” in five studies where the studies did not consider potential confounding factors, such as smoking status, diabetes, socioeconomic status, and educational status, except for age and sex. For detection bias, all studies were rated as having a “definitely low” with regard to appropriate methods for exposure characterization. Of 16 studies were rated as having a “definitely low” risk and 15 as having a “probably low” risk for detection bias in outcome assessment with valid outcome assessments based on ICD-code. For selection bias, 30 studies were rated as having a “definitely low” risk and one as having a “probably low” risk of selection bias due to a clear definition of comparison groups. Of those, 15 studies were rated as having a “probably high” risk of attrition/exclusion bias because of insufficient information to justify assessment, and 16 studies were rated as having a “definitely low” risk of attrition/exclusion bias. All studies were rated as having a “definitely low” risk of bias for the selective reporting of results due to reporting results with sufficient detail to be included in the meta-analysis. Two studies were rated as having a “definitely high” risk of bias for conflict of interest because they reported in their studies, 26 studies were rated as having a “probably high” risk of bias due to a lack of reporting of conflicts of interest, and three studies were rated as having a “definitely low” risk of bias for conflict of interest due to their reported in the studies. In summary, 26 and five studies were categorized as tiers 1 and 2, respectively, for risk of bias with regard to the OHAT criteria, indicating “plausible bias that raises some doubt about the results.”

**Table S4**. Summary of risk of bias of human epidemiological studies using the OHAT risk of bias rating tool for pancreatic cancer.

| **Bias domain** | | **Alguacil et al., 2000** | **Alguacil et al., 2000** | **Bardin et al., 1997** | **Beard et al., 2003** | **Benson et al., 1993** | **De Roos et al., 2000** | **Garabrant et al., 1992** | **Greenberg et al., 2000** | **Hansen et al., 1989** | **Hidajat et al., 2019** | **Kauppinen et al., 1995** | **Kolstad et al., 2000** | **Lerro et al., 2020** | **Li et al., 2006** | **Loomis et al., 2018** | **Lundin et al., 2008** |
| --- | --- | --- | --- | --- | --- | --- | --- | --- | --- | --- | --- | --- | --- | --- | --- | --- | --- |
| **++** | Definitely low risk of bias |
| **+** | Probably low risk of bias |
| **-** | Probably high risk of bias |
| **--** | Definitely high risk of bias |
| **Confounding bias** | |  |  |  |  |  |  |  |  |  |  |  |  |  |  |  |  |
| 1. Did the study design or analysis account for important confounding and modifying variables? **(Key domain)** | | **++** | **++** | **+** | **+** | **+** | **++** | **+** | **+** | **+** | **+** | **++** | **-** | **++** | **+** | **+** | **+** |
| **Detection bias** | |  |  |  |  |  |  |  |  |  |  |  |  |  |  |  |  |
| 1. Can we be confident in the exposure characterization? **(Key domain)** | | **++** | **++** | **++** | **++** | **++** | **++** | **++** | **++** | **++** | **++** | **++** | **++** | **++** | **++** | **++** | **++** |
| 1. Can we be confident in the outcome assessment? **(Key domain)** | | **+** | **+** | **+** | **+** | **+** | **++** | **+** | **+** | **+** | **+** | **++** | **++** | **++** | **++** | **++** | **++** |
| **Selection bias** | |  |  |  |  |  |  |  |  |  |  |  |  |  |  |  |  |
| 1. Did selection of study participants result in appropriate comparison groups? | | **++** | **++** | **++** | **++** | **++** | **++** | **++** | **++** | **++** | **++** | **++** | **++** | **++** | **++** | **++** | **++** |
| **Attrition/Exclusion bias** | |  |  |  |  |  |  |  |  |  |  |  |  |  |  |  |  |
| 1. Were outcome data complete without attrition or exclusion from analysis? | | **++** | **++** | **++** | **++** | **-** | **++** | **-** | **-** | **-** | **-** | **++** | **++** | **++** | **-** | **++** | **++** |
| **Selective reporting bias** | |  |  |  |  |  |  |  |  |  |  |  |  |  |  |  |  |
| 1. Were all measured outcome reported? | | **++** | **++** | **++** | **++** | **++** | **++** | **++** | **++** | **++** | **++** | **++** | **++** | **++** | **++** | **++** | **++** |
| **Other bias** | |  |  |  |  |  |  |  |  |  |  |  |  |  |  |  |  |
| 1. Conflict of interest | | **-** | **-** | **-** | **-** | **-** | **-** | **-** | **-** | **-** | **++** | **-** | **-** | **++** | **-** | **++** | **-** |
| **Summary tier category** | | **T1** | **T1** | **T1** | **T1** | **T1** | **T1** | **T1** | **T1** | **T1** | **T1** | **T1** | **T2** | **T1** | **T1** | **T1** | **T1** |

Abbreviations: T, tier

**Table S4.** Summary of risk of bias of human epidemiological studies using the OHAT risk of bias rating tool for pancreatic cancer.

| **Bias domain** | | **Lynge et al., 2006** | **Marsh et al., 2007** | **Mikoczy et al., 1996** | **Olsen et al., 1997** | **Reul et al., 2016** | **Romundstad et al., 2000** | **Romundstad et al., 2000** | **Saarni et al., 2002** | **Sauni et al., 2017** | **Selenskas et al., 1995** | **Silverstein et al., 1988** | **Steenland et al., 2012** | **Teta et al., 1993** | **Van Barneveld et al., 2004** | **Zhang et al., 2005** |
| --- | --- | --- | --- | --- | --- | --- | --- | --- | --- | --- | --- | --- | --- | --- | --- | --- |
| **++** | Definitely low risk of bias |
| **+** | Probably low risk of bias |
| **-** | Probably high risk of bias |
| **--** | Definitely high risk of bias |
| **Confounding bias** | |  |  |  |  |  |  |  |  |  |  |  |  |  |  |  |
| 1. Did the study design or analysis account for important confounding and modifying variables? **(Key domain)** | | **+** | **+** | **+** | **+** | **+** | **+** | **+** | **-** | **-** | **+** | **-** | **-** | **+** | **+** | **++** |
| **Detection bias** | |  |  |  |  |  |  |  |  |  |  |  |  |  |  |  |
| 1. Can we be confident in the exposure characterization? **(Key domain)** | | **++** | **++** | **++** | **++** | **++** | **++** | **++** | **++** | **++** | **++** | **++** | **++** | **++** | **++** | **++** |
| 1. Can we be confident in the outcome assessment? **(Key domain)** | | **++** | **+** | **++** | **++** | **+** | **++** | **++** | **++** | **+** | **+** | **++** | **++** | **+** | **++** | **+** |
| **Selection bias** | |  |  |  |  |  |  |  |  |  |  |  |  |  |  |  |
| 1. Did selection of study participants result in appropriate comparison groups? | | **++** | **++** | **++** | **++** | **++** | **++** | **++** | **++** | **++** | **++** | **++** | **++** | **++** | **++** | **+** |
| **Attrition/Exclusion bias** | |  |  |  |  |  |  |  |  |  |  |  |  |  |  |  |
| 1. Were outcome data complete without attrition or exclusion from analysis? | | **-** | **-** | **++** | **-** | **++** | **-** | **-** | **-** | **-** | **-** | **-** | **++** | **++** | **++** | **++** |
| **Selective reporting bias** | |  |  |  |  |  |  |  |  |  |  |  |  |  |  |  |
| 1. Were all measured outcome reported? | | **++** | **++** | **++** | **++** | **++** | **++** | **++** | **++** | **++** | **++** | **++** | **++** | **++** | **++** | **++** |
| **Other bias** | |  |  |  |  |  |  |  |  |  |  |  |  |  |  |  |
| 1. Conflict of interest | | **-** | **-** | **-** | **-** | **--** | **-** | **-** | **-** | **--** | **-** | **-** | **-** | **-** | **-** | **-** |
| **Summary tier category** | | **T1** | **T1** | **T1** | **T1** | **T1** | **T1** | **T1** | **T2** | **T2** | **T1** | **T2** | **T2** | **T1** | **T1** | **T1** |

Abbreviations: T, tier

**Table S4A.** Risk of bias of Alguacil et al., 2000, according instructions report based on the OHAT risk of bias tool.

| **Bias domain** | **Risk of Bias** | **Comments** |
| --- | --- | --- |
| **Selection bias**  Did selection of study participants result in appropriate comparison groups? | Definitely low risk of bias | Participants were recruited in 1992 to 1995 from five general hospitals in eastern Spain. Incident cases of pancreatic cancer and hospital controls were prospectively identified and interviewed during the hospital stay. Controls were participants free of pancreatic cancer who had been admitted to the same hospitals with an initial diagnostic suspicion of pancreatic cancer, biliary cancer, or chronic pancreatitis.  Both groups are recruited from the same database. |
| **Confounding bias**  Did the study design or analysis account for important confounding and modifying variables? | Definitely low risk of bias | This study adjusted confounding factors in the models, including age, hospital, smoking, coffee consumption, and alcohol use. In addition, risks for the a priori high-risk occupations were further assessed by duration of exposure and by applying a period analysis. |
| **Attrition/ Exclusion bias**  Were outcome data complete without attrition or exclusion from analysis? | Definitely low risk of bias | The participants included of 185 pancreatic cancer cases and 264 controls. Due to occupational histories were obtained for 164 of the cases, 21 cases were excluded.  There is direct evidence that the exclusion of participants from analyses was adequately addressed, and reasons were documented when participants were removed from the study or excluded from analyses. |
| **Detection bias**  1. Can we be confident in the exposure characterization? | Definitely low risk of bias | Case and control were asked by interviews. Patients were asked if they had ever worked in any of ten activities related to pancreas and biliary cancers. These were pesticide use, handling of petroleum derivatives, the chemical industry, the metal industry, the rubber industry, the graphic arts, jewelry, the manufacture or repair of automobiles, leather tanning, and the textile industry. |
| 2. Can we be confident in the outcome assessment? | Probably low risk of bias | Cases were pancreatic cancer, and controls were participants free of pancreatic cancer who had been admitted to the same hospitals with an initial diagnostic suspicion of pancreatic cancer, biliary cancer, or chronic pancreatitis.  The definition is well-documented. |
| **Selective reporting bias**  Were all measured outcomes reported? | Definitely low risk of bias | The associations were explored separately for men and women. Multivariate-adjusted odds ratio and 95% confidence interval were estimated by unconditional logistic regression.  This outcome provides sufficient detail to be included in the meta-analysis. |
| **Other bias**  Conflict of interest | Probably high risk of bias | Not specified. |

**Table S4B.** Risk of bias of Alguacil et al., 2000, according instructions report based on the OHAT risk of bias tool.

| **Bias domain** | **Risk of Bias** | **Comments** |
| --- | --- | --- |
| **Selection bias**  Did selection of study participants result in appropriate comparison groups? | Definitely low risk of bias | Participants were recruited in 1992 to 1995 from five general hospitals in eastern Spain. Incident cases of pancreatic cancer and hospital controls were prospectively identified and interviewed during the hospital stay. Controls were participants free of pancreatic cancer who had been admitted to the same hospitals with an initial diagnostic suspicion of pancreatic cancer, biliary cancer, or chronic pancreatitis. Both groups are recruited from the same database. |
| **Confounding bias**  Did the study design or analysis account for important confounding and modifying variables? | Definitely low risk of bias | This study adjusted confounding factors in the models, including age, hospital, smoking, coffee consumption, and alcohol use. |
| **Attrition/ Exclusion bias**  Were outcome data complete without attrition or exclusion from analysis? | Definitely low risk of bias | The participants included 185 pancreatic cancer cases and 264 controls. Due to occupational histories were obtained for 164 of the cases and 238 of the controls, 21 cases and 26 controls were excluded.  There is direct evidence that the exclusion of participants from analyses was adequately addressed, and reasons were documented when participants were removed from the study or excluded from analyses. |
| **Detection bias**  1. Can we be confident in the exposure characterization? | Definitely low risk of bias | Case and control were asked by interviews. Patients were asked if they had ever worked in any of ten activities related to pancreas and biliary cancers. These were pesticide use, handling of petroleum derivatives, the chemical industry, the metal industry, the rubber industry, the graphic arts, jewelry, the manufacture or repair of automobiles, leather tanning, and the textile industry. |
| 2. Can we be confident in the outcome assessment? | Probably low risk of bias | Cases were pancreatic cancer, and controls were participants free of pancreatic cancer who had been admitted to the same hospitals with an initial diagnostic suspicion of pancreatic cancer, biliary cancer, or chronic pancreatitis.  The definition is well-documented. |
| **Selective reporting bias**  Were all measured outcomes reported? | Definitely low risk of bias | Multivariate-adjusted odds ratio and 95% confidence interval were estimated by unconditional logistic regression.  This outcome provides sufficient detail to be included in the meta-analysis. |
| **Other bias**  Conflict of interest | Probably high risk of bias | Not specified. |

**Table S4C.** Risk of bias of Bardin et al., 1997, according instructions report based on the OHAT risk of bias tool.

| **Bias domain** | **Risk of Bias** | **Comments** |
| --- | --- | --- |
| **Selection bias**  Did selection of study participants result in appropriate comparison groups? | Definitely low risk of bias | The participant in this study were recruited from the same cohort, which consisted of 46,384 hourly employees who had worked at least three years prior to January 1, 1985, at three auto parts manufacturing facilities in Michigan.  Both groups are recruited from the same database. |
| **Confounding bias**  Did the study design or analysis account for important confounding and modifying variables? | Probably low risk of bias | Controls matched with cases as: controls have lived at least as long as the case and any exposure incurred after this date is not included in cumulative exposure. Additional matching factors included race, sex, plant, and date of birth (±5 years). In addition, years since hire were added to all models as a covariate in order to minimize confounding due to the health-worker effect.  This study considers only a partial list of covariates in the final analysis. |
| **Attrition/ Exclusion bias**  Were outcome data complete without attrition or exclusion from analysis? | Definitely low risk of bias | Participants who had been exposed to machining or synthetic were excluded from the analysis due to uncommon exposure. Exposure to chromium was also excluded from the analysis due to rare exposure.  There is direct evidence that the exclusion of participants from analyses was adequately addressed, and reasons were documented when participants were removed from the study or excluded from analyses. |
| **Detection bias**  1. Can we be confident in the exposure characterization? | Definitely low risk of bias | Autoworkers exposed to metalworking fluids (MWF) were classified as either straight, soluble, or synthetic fluids. Both soluble and synthetic fluids are water-based. Soluble contain petroleum oil emulsified in water, and synthetics contain only synthetic lubricants. Exposure is estimated for each unique plant, department, job, and calendar period in an exposure matrix. For each cell in the matrix, quantitative assessments (mg/m^3^) were made for MWF and classified by type of MWF and metal-working operation.  There is direct evidence that exposure was consistently assessed with the same method. |
| 2. Can we be confident in the outcome assessment? | Probably low risk of bias | Pancreatic cancer cases are defined as those participants for whom pancreatic cancer was listed as the underlying cause of death on the death certificate or as an “other significant condition.”  The definition is well-documented but not defined by a gold standard. |
| **Selective reporting bias**  Were all measured outcomes reported? | Definitely low risk of bias | The outcome was reported as odds ratio and 95% confidence interval by using conditional logistic regression.  This outcome provides sufficient detail to be included in the meta-analysis. |
| **Other bias**  Conflict of interest | Probably high risk of bias | Not specified. |

**Table S4D.** Risk of bias of Beane et al., 2011, according instructions report based on the OHAT risk of bias tool.

| **Bias domain** | **Risk of Bias** | **Comments** |
| --- | --- | --- |
| **Selection bias**  Did selection of study participants result in appropriate comparison groups? | Definitely low risk of bias | The participants in this study were recruited from the Agricultural Health Study (AHS) cohort study that included 57,310 licensed pesticide applicators in Iowa and North Carolina. Applicators were recruited and enrolled in the study during 1993–1997 when they obtained or renewed their licenses to apply restricted-use pesticides.  Both groups are recruited from the same database. |
| **Confounding bias**  Did the study design or analysis account for important confounding and modifying variables? | Definitely low risk of bias | This study was adjusted to confounding factors for race, sex, age, smoking history, alcohol use, education, state of residence, family history of cancer, applicator type, and ever use of other pesticides most highly correlated with atrazine use. |
| **Attrition/ Exclusion bias**  Were outcome data complete without attrition or exclusion from analysis? | Definitely low risk of bias | Of the 57,310 applicators in the AHS, this study excluded 2,337 who provided no or insufficient information on atrazine use to calculate lifetime or intensity-weighted lifetime days, 1,044 with a cancer diagnosis before enrollment, and 267 who had missing or zero person-years.  There is direct evidence that the exclusion of participants from analyses was adequately addressed, and reasons were documented when participants were removed from the study or excluded from analyses. |
| **Detection bias**  1. Can we be confident in the exposure characterization? | Definitely low risk of bias | Exposure assessment in this study used the self-administered questionnaire to assess the exposure, which is available at <http://aghealth.nci.nih.gov/questionnaires.html>. Questionnaire data was used to construct two measures of atrazine use as: 1) lifetime days of use, calculated by multiplying the years of reported atrazine use by the mean number of days per year of use; and 2) intensity-weighted lifetime days of use, calculated by multiplying lifetime days of atrazine use by a measure of exposure intensity based on general handling practices for pesticides.  There is direct evidence that exposure was consistently assessed with the same method. |
| 2. Can we be confident in the outcome assessment? | Probably low risk of bias | Pancreatic cancer was ascertained through linkage to state cancer registries in Iowa and North Carolina.  This study didn’t indicate the gold standard of outcome definition. |
| **Selective reporting bias**  Were all measured outcomes reported? | Definitely low risk of bias | The outcome was reported as relative risk and 95% confidence interval by using Poisson regression.  This outcome provides sufficient detail to be included in the meta-analysis. |
| **Other bias**  Conflict of interest | Probably high risk of bias | Not specified. |

**Table S4E.** Risk of bias of Beard et al., 2003, according instructions report based on the OHAT risk of bias tool.

| **Bias domain** | **Risk of Bias** | **Comments** |
| --- | --- | --- |
| **Selection bias**  Did selection of study participants result in appropriate comparison groups? | Definitely low risk of bias | The exposed cohort was made up of all male staff identified by a search of New South Wales (NSW) government records as having worked as field officers or laboratory staff for the NSW Board of Tick Control at any time since 1935. The control cohort was made up of all male staff identified by local governments from the same region as having worked as outdoor field officers at any time since 1935. Both groups are recruited from the same region. |
| **Confounding bias**  Did the study design or analysis account for important confounding and modifying variables? | Probably low risk of bias | This study adjusted confounding factors for log age with likelihood ratio confidence limits, 10-year exposure lag, the year of death occurring before or after 1960, and smoking.  This study considers only a partial list of covariates in the final analysis. |
| **Attrition/ Exclusion bias**  Were outcome data complete without attrition or exclusion from analysis? | Definitely low risk of bias | The participants with incomplete information were excluded from the analysis.  There is direct evidence that the exclusion of participants from analyses was adequately addressed, and reasons were documented when participants were removed from the study or excluded from analyses. |
| **Detection bias**  1. Can we be confident in the exposure characterization? | Definitely low risk of bias | The exposure assessment in this study is pesticide use. A participant’s period of employment was used to estimate both the type of chemicals he was likely to have been exposed to and the duration of this exposure. This was categorized into exposure groups: “All” equal to any employment during a particular period, “Dose 0” related to exposed participants not yet past the 10-year exposure lag, “Dose 1” equal to < 5 years of employment, “Dose 2” equal to ≥ 5 to < 15 years of employment, and “Dose 3” equal to ≥ 15 years of employment.  There is direct evidence that exposure was consistently assessed with the same method. |
| 2. Can we be confident in the outcome assessment? | Probably low risk of bias | Pancreatic cancer was defined by the cancer registry.  This study didn’t indicate the gold standard of outcome definition. |
| **Selective reporting bias**  Were all measured outcomes reported? | Definitely low risk of bias | The outcome was reported as standardized incidence ratio and 95% confidence interval by using Poisson regression.  This outcome provides sufficient detail to be included in the meta-analysis. |
| **Other bias**  Conflict of interest | Probably high risk of bias | Not specified. |

**Table S4F**. Risk of bias of Benson et al., 1993, according instructions report based on the OHAT risk of bias tool.

| **Bias domain** | **Risk of Bias** | **Comments** |
| --- | --- | --- |
| **Selection bias**  Did selection of study participants result in appropriate comparison groups? | Definitely low risk of bias | The study cohort consisted of 278 male workers who had ever been assigned to the chlorohydrin unit at Union Carbide’s South Charleston plant between January 1, 1940, and December 31, 1967. Both exposure and non-exposure groups are recruited from the same region. |
| **Confounding bias**  Did the study design or analysis account for important confounding and modifying variables? | Probably low risk of bias | This study adjusted confounding factors for age, calendar period, and interval since assignments.  This study considers only a partial list of covariates in the final analysis. |
| **Attrition/ Exclusion bias**  Were outcome data complete without attrition or exclusion from analysis? | Probably high risk of bias | Not specified. |
| **Detection bias**  1. Can we be confident in the exposure characterization? | Definitely low risk of bias | Exposure assessment in this study was defined as male workers who had been assigned to the chlorohydrin unit at Union Carbide’s South Charleston plant.  There is direct evidence that exposure was consistently assessed with the same method. |
| 2. Can we be confident in the outcome assessment? | Probably low risk of bias | Vital status for the chlorohydrin unit workers was obtained from company records and matched with the United States National Death Index.  This study didn’t indicate the gold standard of outcome definition. |
| **Selective reporting bias**  Were all measured outcomes reported? | Definitely low risk of bias | The outcome was reported as relative risk and 95% confidence interval.  This outcome provides sufficient detail to be included in the meta-analysis. |
| **Other bias**  Conflict of interest | Probably high risk of bias | Not specified. |

**Table S4G.** Risk of bias of De Roos et al., 2000, according instructions report based on the OHAT risk of bias tool.

| **Bias domain** | **Risk of Bias** | **Comments** |
| --- | --- | --- |
| **Selection bias**  Did selection of study participants result in appropriate comparison groups? | Definitely low risk of bias | The study population was recruited from the AHS cohort study in Iowa and North Carolina, which included 57,311 private and commercial applicators who were licensed to apply restricted-use pesticides at the time of enrollment. Recruitment of the applicators occurred between 1993 and 1997. Both exposure and non-exposure groups are recruited using the same databases. |
| **Confounding bias**  Did the study design or analysis account for important confounding and modifying variables? | Definitely low risk of bias | This study was adjusted confounding factors for age at enrollment, education, pack-years of cigarette smoking, alcohol consumption in the past year, family history of cancer in first-degree relatives, state of residence, and five pesticides for which cumulative-exposure-day variables were with acetic acid, alachlor, atrazine, metolachlor, and trifluralin. |
| **Attrition/ Exclusion bias**  Were outcome data complete without attrition or exclusion from analysis? | Definitely low risk of bias | Persons whose first primary cancer occurred before the time of enrollment, participants who were lost to follow-up or otherwise did not contribute any person-time, and applicators who did not provide any information on age or whether they had ever used glyphosate were excluded from the analysis. |
| **Detection bias**  1. Can we be confident in the exposure characterization? | Definitely low risk of bias | This study uses a questionnaire to collect information from applicators. Glyphosate exposure metrics for the analysis are defined as: a) ever personally mixed or applied products containing glyphosate; b) cumulative lifetime days of use or cumulative exposure days; and c) intensity-weighted cumulative exposure days.  There is direct evidence that exposure was consistently assessed with the same method. |
| 2. Can we be confident in the outcome assessment? | Definitely low risk of bias | Pancreatic cancer is defined as any case with code 157 of the International Classification of Diseases (9^th^ revision).  The definition is well-documented. |
| **Selective reporting bias**  Were all measured outcomes reported? | Definitely low risk of bias | The outcome was reported as relative risk and 95% confidence interval.  This outcome provides sufficient detail to be included in the meta-analysis. |
| **Other bias**  Conflict of interest | Probably high risk of bias | Not specified. |

**Table S4H.** Risk of bias of Garabrant et al., 1992, according instructions report based on the OHAT risk of bias tool.

| **Bias domain** | **Risk of Bias** | **Comments** |
| --- | --- | --- |
| **Selection bias**  Did selection of study participants result in appropriate comparison groups? | Definitely low risk of bias | The study population was recruited from the same plant. Cases were identified by the company through ongoing surveillance of deaths among the cohort of employees that met the following criteria: all males employed in the plant under study at any time during the period from January 1, 1948, to August 1, 1971. Controls were identified from the roster of plant employees by preparing a list of those who were matched to the case patient on sex and race and who were alive at the age at which the pancreatic cancer was diagnosed.  There is indirect evidence that cases and controls were similar, which is they were recruited from the same eligible population, same area, and similar age. |
| **Confounding bias**  Did the study design or analysis account for important confounding and modifying variables? | Probably low risk of bias | This study adjusted potential confounding factors for age and sex, but other factors didn’t state which factors were controlled.  This study considers only a partial list of covariates in the final analysis. |
| **Attrition/ Exclusion bias**  Were outcome data complete without attrition or exclusion from analysis? | Probably high risk of bias | Not specified. |
| **Detection bias**  1. Can we be confident in the exposure characterization? | Definitely low risk of bias | The exposure assessment in this study was a structured interview by telephone. The information that was interviewed in this study was: religion, education, medical history, use of medications, smoking habits, family medical history, diet, occupational history, and past chemical exposure.  There is direct evidence that exposure was consistently assessed with the same method. |
| 2. Can we be confident in the outcome assessment? | Probably low risk of bias | A pancreatic cancer case was diagnosed with histologic confirmation.  There is no gold standard for evaluating outcomes. |
| **Selective reporting bias**  Were all measured outcomes reported? | Definitely low risk of bias | The outcome was reported as relative risk and 95% confidence interval.  This outcome provides sufficient detail to be included in the meta-analysis. |
| **Other bias**  Conflict of interest | Probably high risk of bias | Not specified. |

**Table S4I.** Risk of bias of Greenberg et al., 2000, according instructions report based on the OHAT risk of bias tool.

| **Bias domain** | **Risk of Bias** | **Comments** |
| --- | --- | --- |
| **Selection bias**  Did selection of study participants result in appropriate comparison groups? | Definitely low risk of bias | The study population was recruited from a base file of 29,139 male employees of Union Carbide Corporation chemical production plants who had ever been employed at any of three Union Carbide Corporation plants in the Kanawha Valley of West Virginia (the two production plants and a technical center) between 1940 and 1978.  There is direct evidence that cases and controls were similar, which is they were recruited from the same eligible population, same area, and similar age. |
| **Confounding bias**  Did the study design or analysis account for important confounding and modifying variables? | Probably low risk of bias | This study adjusted potential confounding factors for age, calendar period, and interval since hire.  This study considers only a partial list of covariates in the final analysis. |
| **Attrition/ Exclusion bias**  Were outcome data complete without attrition or exclusion from analysis? | Probably high risk of bias | Not specified. |
| **Detection bias**  1. Can we be confident in the exposure characterization? | Definitely low risk of bias | The exposure assessment in this study was an assessment of exposure to ethylene oxide, which was obtained by interviews and by reference to available records.  There is direct evidence that exposure was consistently assessed with the same method. |
| 2. Can we be confident in the outcome assessment? | Probably low risk of bias | The outcome was identified by the death certificate.  No gold standard to identify an outcome. |
| **Selective reporting bias**  Were all measured outcomes reported? | Definitely low risk of bias | The outcome was reported as relative risk and 95% confidence interval.  This outcome provides sufficient detail to be included in the meta-analysis. |
| **Other bias**  Conflict of interest | Probably high risk of bias | Not specified. |

**Table S4J.** Risk of bias of Hidajat et al., 2019, according instructions report based on the OHAT risk of bias tool.

| **Bias domain** | **Risk of Bias** | **Comments** |
| --- | --- | --- |
| **Selection bias**  Did selection of study participants result in appropriate comparison groups? | Definitely low risk of bias | The study population was recruited from a cohort of male UK rubber factory workers aged 35 years or older as of 1 February 1967 and followed up for mortality to December 2015.  There is direct evidence that cases and controls were similar, which is they were recruited from the same eligible population, same area, and similar age. |
| **Confounding bias**  Did the study design or analysis account for important confounding and modifying variables? | Probably low risk of bias | This study adjusted potential confounding factors for age and birth year.  This study considers only a partial list of covariates in the final analysis. |
| **Attrition/ Exclusion bias**  Were outcome data complete without attrition or exclusion from analysis? | Probably high risk of bias | Not specified. |
| **Detection bias**  1. Can we be confident in the exposure characterization? | Definitely low risk of bias | The exposure assessment was based on estimates from the database of exposures in the rubber manufacturing industry in Europe. The primary analysis assumed all participants remained in the same factory department. Lifetime cumulative exposure to rubber dust, rubber fumes and N-nitrosamines was calculated for each worker based on the assumed number of years worked and department.  There is direct evidence that exposure was consistently assessed with the same method. |
| 2. Can we be confident in the outcome assessment? | Probably low risk of bias | Outcomes are mortality from cancers previously associated with the rubber industry: all cancers, cancers of the bladder, lung, stomach, multiple myeloma, leukemia, larynx, esophagus, prostate, non-Hodgkin’s lymphoma, pancreas, brain, liver, and in situ, benign or unknown behavior neoplasms.  No gold standard to identify an outcome. |
| **Selective reporting bias**  Were all measured outcomes reported? | Definitely low risk of bias | The outcome was reported as hazard ratio and 95% confidence interval.  This outcome provides sufficient detail to be included in the meta-analysis. |
| **Other bias**  Conflict of interest | Definitely low risk of bias | The authors declared that there was no conflict of interest. |

**Table S4K**. Risk of bias of Kauppinen et al., 1995, according instructions report based on the OHAT risk of bias tool.

| **Bias domain** | **Risk of Bias** | **Comments** |
| --- | --- | --- |
| **Selection bias**  Did selection of study participants result in appropriate comparison groups? | Definitely low risk of bias | The study population was recruited from the Finnish Cancer Register. Cases were defined as those who had contracted primary exocrine pancreatic cancer in Finland in 1984–1987 at the age of 40–74 years. Controls were defined as participants who had contracted stomach cancer, colon cancer, or rectal cancer, chosen by using similar age and period criteria as for the case series.  There is direct evidence that cases and controls were similar, which is they were recruited from the same eligible population, same area, and similar age. |
| **Confounding bias**  Did the study design or analysis account for important confounding and modifying variables? | Definitely low risk of bias | This study was adjusted for potential confounding factors for age, gender, smoking in the 1960s, history of diabetes mellitus, and alcohol consumption in the 1960s. |
| **Attrition/ Exclusion bias**  Were outcome data complete without attrition or exclusion from analysis? | Definitely low risk of bias | Patients with histologically confirmed cases of endocrine pancreatic cancer, non-respondents, participants with very incomplete work histories, participants with diagnosed pancreatitis, and administrators and managers were excluded. |
| **Detection bias**  1. Can we be confident in the exposure characterization? | Definitely low risk of bias | Exposure was defined as exposure to chemical and physical agents. The exposures were classified as none, light, moderate, and heavy. Assignment to the class “heavy” required at least 10 years of work in conditions where the level of exposure was “high.” “Moderate” refers to less than 10 years’ employment in “high” exposure or at least 10 years’ employment in “low” exposure. “Light” refers to less than 10 years’ employment in “low” exposure. This study considered the level “high” if it was likely to have exceeded 50% of either the threshold limit value or of the biological exposure index adopted by the American Conference of Governmental Industrial Hygienists.  There is direct evidence that exposure was consistently assessed with the same method. |
| 2. Can we be confident in the outcome assessment? | Definitely low risk of bias | Pancreatic cancer is defined as any case with code 157 of the International Classification of Diseases (9^th^ revision).  The definition is well-documented. |
| **Selective reporting bias**  Were all measured outcomes reported? | Definitely low risk of bias | The outcome was reported as odds ratio and 95% confidence interval.  This outcome provides sufficient detail to be included in the meta-analysis. |
| **Other bias**  Conflict of interest | Probably high risk of bias | Not specified. |

**Table S4L.** Risk of bias of Kolstad et al., 2000, according instructions report based on the OHAT risk of bias tool.

| **Bias domain** | **Risk of Bias** | **Comments** |
| --- | --- | --- |
| **Selection bias**  Did selection of study participants result in appropriate comparison groups? | Definitely low risk of bias | The study population was recruited from the 53,847 male workers employed between 1964 and 1988 and alive on January 1, 1970.  There is direct evidence that cases and controls were similar, which is they were recruited from the same eligible population, same area, and similar age. |
| **Confounding bias**  Did the study design or analysis account for important confounding and modifying variables? | Probably high risk of bias | The study was adjusted by age stratification.  Other confounding factors weren’t adjusted in the model. |
| **Attrition/ Exclusion bias**  Were outcome data complete without attrition or exclusion from analysis? | Definitely low risk of bias | The company with unknown to the dealers were excluded. |
| **Detection bias**  1. Can we be confident in the exposure characterization? | Definitely low risk of bias | Exposure was defined as probable low exposure to styrene (employees of companies with less than 50% of the workers involved with reinforced plastics) and probable high exposure (employees of companies with 50% or more of the workers involved with reinforced plastics). The duration of employment was estimated from the payments recorded for each employee in the pension fund.  There is direct evidence that exposure was consistently assessed with the same method. |
| 2. Can we be confident in the outcome assessment? | Definitely low risk of bias | Pancreatic cancer is defined as any case with code 157 of the International Classification of Diseases (7^th^ revision).  The definition is well-documented. |
| **Selective reporting bias**  Were all measured outcomes reported? | Definitely low risk of bias | The outcome was reported as relative risk and 95% confidence interval.  This outcome provides sufficient detail to be included in the meta-analysis. |
| **Other bias**  Conflict of interest | Probably high risk of bias | Not specified. |

**Table S4M.** Risk of bias of Lerro et al., 2020, according instructions report based on the OHAT risk of bias tool.

| **Bias domain** | **Risk of Bias** | **Comments** |
| --- | --- | --- |
| **Selection bias**  Did selection of study participants result in appropriate comparison groups? | Definitely low risk of bias | The study population was recruited from the AHS cohort that includes 57,310 licensed pesticide applicators enrolled during 1993–1997 in Iowa and North Carolina. A follow-up interview was conducted during 1999–2005.  There is direct evidence that cases and controls were similar, which is they were recruited from the same eligible population, same area, and similar age. |
| **Confounding bias**  Did the study design or analysis account for important confounding and modifying variables? | Definitely low risk of bias | This study was adjusted for potential confounding factors for age, race, state, applicator type, education, imazethapyr, smoking, and family history of cancer. |
| **Attrition/ Exclusion bias**  Were outcome data complete without attrition or exclusion from analysis? | Definitely low risk of bias | Participants who enrolled out of state, those diagnosed with cancer prior to enrollment, and those with missing dicamba intensity-weighed days at enrollment were excluded. |
| **Detection bias**  1. Can we be confident in the exposure characterization? | Definitely low risk of bias | The questionnaire was used to assess the exposure. Participants provided information on the duration (years) and frequency (means days/year) of dicamba use.  There is direct evidence that exposure was consistently assessed with the same method. |
| 2. Can we be confident in the outcome assessment? | Definitely low risk of bias | Pancreatic cancer is defined as any case with code C25 of the International Classification of Diseases for Oncology (3^rd^ revision).  The definition is well-documented. |
| **Selective reporting bias**  Were all measured outcomes reported? | Definitely low risk of bias | The outcome was reported as relative risk and 95% confidence interval.  This outcome provides sufficient detail to be included in the meta-analysis. |
| **Other bias**  Conflict of interest | Definitely low risk of bias | The authors declared that there was no conflict of interest. |

**Table S4N.** Risk of bias of Li et al., 2006, according instructions report based on the OHAT risk of bias tool.

| **Bias domain** | **Risk of Bias** | **Comments** |
| --- | --- | --- |
| **Selection bias**  Did selection of study participants result in appropriate comparison groups? | Definitely low risk of bias | The study population included 267,400 female employees in 526 factories in the Shanghai Textile Industry Bureau (STIB) who had been enrolled from October 1989 to October 1991 in a randomized trial of breast self-examination (BSE).  There is direct evidence that cases and controls were similar, which is they were recruited from the same eligible population, same area, and similar age. |
| **Confounding bias**  Did the study design or analysis account for important confounding and modifying variables? | Probably low risk of bias | This study adjusted potential confounding factors for age and smoking status.  This study considers only a partial list of covariates in the final analysis. |
| **Attrition/ Exclusion bias**  Were outcome data complete without attrition or exclusion from analysis? | Probably high risk of bias | Not specified. |
| **Detection bias**  1. Can we be confident in the exposure characterization? | Definitely low risk of bias | The questionnaire was used to assess the exposure. A complete occupational history, including employment dates, workshop, and task description in the textile industry, was collected for each of the study participants by 30 trained interviewers by abstracting the relevant information from employment records or interviewing the woman’s supervisor or co-workers. The woman was interviewed by telephone or in-person, following the same study protocol.  There is direct evidence that exposure was consistently assessed with the same method. |
| 2. Can we be confident in the outcome assessment? | Definitely low risk of bias | Pancreatic cancer is defined as any case with code 157.0, 157.1, 157.2, or 157.9 of the International Classification of Diseases (9^th^ revision).  The definition is well-documented. |
| **Selective reporting bias**  Were all measured outcomes reported? | Definitely low risk of bias | The outcome was reported as hazard ratio and 95% confidence interval.  This outcome provides sufficient detail to be included in the meta-analysis. |
| **Other bias**  Conflict of interest | Probably high risk of bias | Not specified. |

**Table S4O.** Risk of bias of Loomis et al., 2018, according instructions report based on the OHAT risk of bias tool.

| **Bias domain** | **Risk of Bias** | **Comments** |
| --- | --- | --- |
| **Selection bias**  Did selection of study participants result in appropriate comparison groups? | Definitely low risk of bias | The study population included workers in the reinforced plastics industry in Denmark, Finland, Italy (two centers), Norway, Sweden, and the United Kingdom (two centers).  There is direct evidence that exposed and unexposed were similar, which is they were recruited from the same eligible population, same area, and similar age. |
| **Confounding bias**  Did the study design or analysis account for important confounding and modifying variables? | Probably low risk of bias | This study adjusted potential confounding factor for age.  This study considers only a partial list of covariates in the final analysis. |
| **Attrition/ Exclusion bias**  Were outcome data complete without attrition or exclusion from analysis? | Definitely low risk of bias | This study excluded the data from the Norway cohort and did not add new mortality data from the two cohorts whose follow-up had been extended in order to avoid large variations in the time period of observation. |
| **Detection bias**  1. Can we be confident in the exposure characterization? | Definitely low risk of bias | An exposure group was defined as a laminator, production workers with mixed tasks or in small plants with no fixed job titles, and workers who regularly entered areas where styrene was handled but were not involved in manual lamination were classified as exposed to styrene. The duration of exposure was estimated as the time employed in exposed jobs, which may have begun after the start of employment. Quantitative exposure to styrene was estimated from approximately 11,600 personal measurements of styrene in the air in the years 1970–1990.  There is direct evidence that exposure was consistently assessed with the same method. |
| 2. Can we be confident in the outcome assessment? | Definitely low risk of bias | Pancreatic cancer is defined as any case with code 157 of the International Classification of Diseases (8^th^ revision).  The definition is well-documented. |
| **Selective reporting bias**  Were all measured outcomes reported? | Definitely low risk of bias | The outcome was reported as relative risk and 95% confidence interval.  This outcome provides sufficient detail to be included in the meta-analysis. |
| **Other bias**  Conflict of interest | Definitely low risk of bias | The authors declared that there was no conflict of interest. |

**Table S4P.** Risk of bias of Lundin et al., 2008, according instructions report based on the OHAT risk of bias tool.

| **Bias domain** | **Risk of Bias** | **Comments** |
| --- | --- | --- |
| **Selection bias**  Did selection of study participants result in appropriate comparison groups? | Definitely low risk of bias | The study population included employees of a 3M company plant located in Cottage Grove, Minnesota, where ammonium perfluorooctanoate production.  There is direct evidence that exposed and unexposed were similar, which is they were recruited from the same eligible population, same area, and similar age. |
| **Confounding bias**  Did the study design or analysis account for important confounding and modifying variables? | Probably low risk of bias | This study adjusted for potential confounding factors for age, sex, and year of birth.  This study considers only a partial list of covariates in the final analysis. |
| **Attrition/ Exclusion bias**  Were outcome data complete without attrition or exclusion from analysis? | Definitely low risk of bias | The participants who had short-term work experience were excluded. |
| **Detection bias**  1. Can we be confident in the exposure characterization? | Definitely low risk of bias | They used work history records and expert historical knowledge of the manufacturing process to assess the exposure. An expert panel of veteran workers and plant industrial hygienists reviewed job titles and administrative department codes by year to determine where the perfluorochemical production, or the development of perfluorochemical products, took place over the history of the facility. Cumulative exposure weighed exposure calculated on duration of employment and qualitatively-specified exposure intensity, which categorized into three groups as: 1 year (36,499 exposure-days), 1–4.9 years (36,500–182,499 exposure-days) and 5 or more years (182,500 exposure-days) of employment in a job.  There is direct evidence that exposure was consistently assessed with the same method. |
| 2. Can we be confident in the outcome assessment? | Definitely low risk of bias | Pancreatic cancer death was coded by the International Classification of Disease.  The definition is well-documented. |
| **Selective reporting bias**  Were all measured outcomes reported? | Definitely low risk of bias | The outcome was reported as hazard ratio and 95% confidence interval.  This outcome provides sufficient detail to be included in the meta-analysis. |
| **Other bias**  Conflict of interest | Probably high risk of bias | Not specified. |

**Table S4Q.** Risk of bias of Lynge et al., 2006, according instructions report based on the OHAT risk of bias tool.

| **Bias domain** | **Risk of Bias** | **Comments** |
| --- | --- | --- |
| **Selection bias**  Did selection of study participants result in appropriate comparison groups? | Definitely low risk of bias | The study population included all laundry and dry-cleaning workers from the 1970 censuses in Denmark, Finland, Norway, and Sweden. Cases were identified using a combined topography and morphology code from the International Classification of Diseases for Oncology. Controls were randomly selected from the cohort using frequency match by country, sex, 5-year age group, and 5-year calendar period at the time of the diagnosis of the case.  There is direct evidence that exposed and unexposed were similar, which is they were recruited from the same eligible population, same area, and similar age. |
| **Confounding bias**  Did the study design or analysis account for important confounding and modifying variables? | Probably low risk of bias | Controls were randomly selected from the cohort using frequency match by country, sex, 5-year age group, and 5-year calendar period at the time of the diagnosis of the case. This study was adjusted for potential confounding factors for smoking and alcohol use.  This study considers only a partial list of covariates in the final analysis. |
| **Attrition/ Exclusion bias**  Were outcome data complete without attrition or exclusion from analysis? | Probably high risk of bias | Not specified. |
| **Detection bias**  1. Can we be confident in the exposure characterization? | Definitely low risk of bias | Exposure was assessed by a blinded personal telephone interview. The questionnaire asked about occupational tasks in 1970, and if this was dry cleaning, then about length of employment in the shop, size of work force, solvents used, and smoking and drinking habits.    There is direct evidence that exposure was consistently assessed with the same method. |
| 2. Can we be confident in the outcome assessment? | Definitely low risk of bias | Pancreatic cancer death was coded by the International Classification of Disease for Oncology.  The definition is well-documented. |
| **Selective reporting bias**  Were all measured outcomes reported? | Definitely low risk of bias | The outcome was reported as relative risk and 95% confidence interval.  This outcome provides sufficient detail to be included in the meta-analysis. |
| **Other bias**  Conflict of interest | Probably high risk of bias | Not specified. |

**Table S4R.** Risk of bias of Marsh et al., 2007, according instructions report based on the OHAT risk of bias tool.

| **Bias domain** | **Risk of Bias** | **Comments** |
| --- | --- | --- |
| **Selection bias**  Did selection of study participants result in appropriate comparison groups? | Definitely low risk of bias | The participants included all male employees with full-time work experience at any of three the United States plants (Fortier, Louisiana, Kalamazoo, Michigan, Warners, New Jersey, and Botlek).  There is direct evidence that exposed and unexposed were similar, which is they were recruited from the same eligible population, same area, and similar age. |
| **Confounding bias**  Did the study design or analysis account for important confounding and modifying variables? | Probably low risk of bias | The study was adjusted for age and year of birth.  Other confounding factors weren’t adjusted in the model. |
| **Attrition/ Exclusion bias**  Were outcome data complete without attrition or exclusion from analysis? | Probably high risk of bias | Not specified. |
| **Detection bias**  1. Can we be confident in the exposure characterization? | Definitely low risk of bias | Exposure was defined as acrylamide exposure to workers who had a cumulative exposure value greater than 0.001 mg/m^3^ -years, the approximate equivalent of a 1-day mean concentration exposure to the permissible exposure limit of 0.3 mg/m^3^ in effect at that time.  There is direct evidence that exposure was consistently assessed with the same method. |
| 2. Can we be confident in the outcome assessment? | Probably low risk of bias | Pancreatic cancer death was coded by the nosologist to the underlying cause of death using the 8^th^ revision rules of the International Classification of Disease with code 157.  The definition is well-documented. |
| **Selective reporting bias**  Were all measured outcomes reported? | Definitely low risk of bias | The outcome was reported as relative risk and 95% confidence interval.  This outcome provides sufficient detail to be included in the meta-analysis. |
| **Other bias**  Conflict of interest | Probably high risk of bias | Not specified. |

**Table S4S.** Risk of bias of Mikoczy et al., 1996, according instructions report based on the OHAT risk of bias tool.

| **Bias domain** | **Risk of Bias** | **Comments** |
| --- | --- | --- |
| **Selection bias**  Did selection of study participants result in appropriate comparison groups? | Definitely low risk of bias | The participants included employees who had been employed for at least six months during the period 1900–1989 in three Swedish leather tanneries. Cases were diagnosed with pancreatic cancer. Controls were randomly selected from those being followed up at the age of onset of each case.  There is direct evidence that cases and controls were similar, which is they were recruited from the same eligible population, same area, and similar age. |
| **Confounding bias**  Did the study design or analysis account for important confounding and modifying variables? | Probably low risk of bias | Cases and controls were matched by plant and sex. This study was adjusted potential confounding factor for age at risk, sex, and plant.  This study considers only a partial list of covariates in the final analysis. |
| **Attrition/ Exclusion bias**  Were outcome data complete without attrition or exclusion from analysis? | Definitely low risk of bias | This study considers the latency and induction period for excluding participants from analysis during the first 10 years since the start of employment, and non-eligible matched cases and controls were excluded. |
| **Detection bias**  1. Can we be confident in the exposure characterization? | Definitely low risk of bias | Exposure was identified by the work history for each case and control, which was investigated by an occupational hygienist with the assistance of expert panels including former long-term employees with good knowledge of the employees and the production.  There is direct evidence that exposure was consistently assessed with the same method. |
| 2. Can we be confident in the outcome assessment? | Definitely low risk of bias | Pancreatic cancer death was coded by the International Classification of Disease (7^th^ revision).  The definition is well-documented. |
| **Selective reporting bias**  Were all measured outcomes reported? | Definitely low risk of bias | The outcome was reported as odds ratio and 95% confidence interval.  This outcome provides sufficient detail to be included in the meta-analysis. |
| **Other bias**  Conflict of interest | Probably high risk of bias | Not specified. |

**Table S4T.** Risk of bias of Olsen et al., 1997, according instructions report based on the OHAT risk of bias tool.

| **Bias domain** | **Risk of Bias** | **Comments** |
| --- | --- | --- |
| **Selection bias**  Did selection of study participants result in appropriate comparison groups? | Definitely low risk of bias | The participants were included in the population of two ethylene oxide production plants (with each having areas where ethylene chlorohydrin was produced) at the Texas Free-port site. The eligible participant must have been a male employee who worked for at least one month at a job that was considered to have been likely to be in the area of ethylene or propylene chlorohydrin production.  There is direct evidence that exposed and unexposed were similar, which is they were recruited from the same eligible population, same area, and similar age. |
| **Confounding bias**  Did the study design or analysis account for important confounding and modifying variables? | Probably low risk of bias | This study adjusted potential confounding factors for age, calendar period, manufacturing location, and period of employment.  This study considers only a partial list of covariates in the final analysis. |
| **Attrition/ Exclusion bias**  Were outcome data complete without attrition or exclusion from analysis? | Probably high risk of bias | Not specified. |
| **Detection bias**  1. Can we be confident in the exposure characterization? | Definitely low risk of bias | The exposure assessment was conducted by evaluating the employees who worked for at least one month at a job that was considered to have been likely to be in the area of ethylene or propylene chlorohydrin production with the duration of employment.  There is direct evidence that exposure was consistently assessed with the same method. |
| 2. Can we be confident in the outcome assessment? | Definitely low risk of bias | The outcome was specified in accordance with the International Classification of Disease rules at the time of death. |
| **Selective reporting bias**  Were all measured outcomes reported? | Definitely low risk of bias | The outcome was reported as relative risk and 95% confidence interval.  This outcome provides sufficient detail to be included in the meta-analysis. |
| **Other bias**  Conflict of interest | Probably high risk of bias | Not specified. |

**Table S4U.** Risk of bias of Reul et al., 2016, according instructions report based on the OHAT risk of bias tool.

| **Bias domain** | **Risk of Bias** | **Comments** |
| --- | --- | --- |
| **Selection bias**  Did selection of study participants result in appropriate comparison groups? | Definitely low risk of bias | The participants were recruited from women employed by the Shanghai Textile Industrial Bureau.  There is direct evidence that exposed and unexposed were similar, which is they were recruited from the same eligible population, same area, and similar age. |
| **Confounding bias**  Did the study design or analysis account for important confounding and modifying variables? | Probably low risk of bias | This study adjusted potential confounding factors for age and smoking status.  This study considers only a partial list of covariates in the final analysis. |
| **Attrition/ Exclusion bias**  Were outcome data complete without attrition or exclusion from analysis? | Definitely low risk of bias | Participants with missing work history were excluded. |
| **Detection bias**  1. Can we be confident in the exposure characterization? | Definitely low risk of bias | The exposure was assessed by the job exposure matrix. |
| 2. Can we be confident in the outcome assessment? | Probably low risk of bias | Pancreatic cancer was identified by medical image, histology, surgical reports, cytology, clinical history and physical examination, and death certificates. |
| **Selective reporting bias**  Were all measured outcomes reported? | Definitely low risk of bias | The outcome was reported as hazard ratio and 95% confidence interval.  This outcome provides sufficient detail to be included in the meta-analysis. |
| **Other bias**  Conflict of interest | Definitely high risk of bias | The authors declared that there was a potential conflict of interest. |

**Table S4V.** Risk of bias of Romundstad et al., 2000, according instructions report based on the OHAT risk of bias tool.

| **Bias domain** | **Risk of Bias** | **Comments** |
| --- | --- | --- |
| **Selection bias**  Did selection of study participants result in appropriate comparison groups? | Definitely low risk of bias | The participants were recruited from the six Norwegian aluminum smelters.  There is direct evidence that exposed and unexposed were similar, which is they were recruited from the same eligible population, same area, and similar age. |
| **Confounding bias**  Did the study design or analysis account for important confounding and modifying variables? | Probably low risk of bias | This study adjusted potential confounding factors for age and smoking status.  This study considers only a partial list of covariates in the final analysis. |
| **Attrition/ Exclusion bias**  Were outcome data complete without attrition or exclusion from analysis? | Probably high risk of bias | Not specified. |
| **Detection bias**  1. Can we be confident in the exposure characterization? | Definitely low risk of bias | Cumulative exposure was used as an indicator of individual dose and calculated for each person-year under observation as the product of the exposure intensity and duration summed for all jobs held. Unexposed is defined as 1 exposure category per person-time. Exposed is defined as person-time constituted by 3 exposure categories according to the number of expected cases in each exposure category. |
| 2. Can we be confident in the outcome assessment? | Definitely low risk of bias | Pancreatic cancer is defined as any case with code 157 of the International Classification of Diseases (7^th^ revision).  The definition is well-documented. |
| **Selective reporting bias**  Were all measured outcomes reported? | Definitely low risk of bias | The outcome was reported as relative risk and 95% confidence interval.  This outcome provides sufficient detail to be included in the meta-analysis. |
| **Other bias**  Conflict of interest | Probably high risk of bias | Not specified. |

**Table S4W.** Risk of bias of Romundstad et al., 2000, according instructions report based on the OHAT risk of bias tool.

| **Bias domain** | **Risk of Bias** | **Comments** |
| --- | --- | --- |
| **Selection bias**  Did selection of study participants result in appropriate comparison groups? | Definitely low risk of bias | The participants were recruited from the two Norwegian aluminum smelters.  There is direct evidence that exposed and unexposed were similar, which is they were recruited from the same eligible population, same area, and similar age. |
| **Confounding bias**  Did the study design or analysis account for important confounding and modifying variables? | Probably low risk of bias | This study adjusted potential confounding factors for age and smoking status.  This study considers only a partial list of covariates in the final analysis. |
| **Attrition/ Exclusion bias**  Were outcome data complete without attrition or exclusion from analysis? | Probably high risk of bias | Not specified. |
| **Detection bias**  1. Can we be confident in the exposure characterization? | Definitely low risk of bias | The exposure was assessed by personal measurement. Exposure to polycyclic aromatic hydrocarbons was an exposure in this study. |
| 2. Can we be confident in the outcome assessment? | Definitely low risk of bias | Pancreatic cancer is defined as any case with code 157 of the International Classification of Diseases (7^th^ revision).  The definition is well-documented. |
| **Selective reporting bias**  Were all measured outcomes reported? | Definitely low risk of bias | The outcome was reported as relative risk and 95% confidence interval.  This outcome provides sufficient detail to be included in the meta-analysis. |
| **Other bias**  Conflict of interest | Probably high risk of bias | Not specified. |

**Table S4X.** Risk of bias of Saarni et al., 2002, according instructions report based on the OHAT risk of bias tool.

| **Bias domain** | **Risk of Bias** | **Comments** |
| --- | --- | --- |
| **Selection bias**  Did selection of study participants result in appropriate comparison groups? | Definitely low risk of bias | The participants were all male seafarers who had worked on board Finnish ships anytime during the period 1960–1980 and who had not died before January 1, 1967, as identified from the files of the Seamen’s Pension Fund. Cases were diagnosed after the date of first employment registered in the Seamen’s Pension Fund or on January 1, 1967. Controls were randomly selected from the same seafarer cohort. The controls had to have the same year of birth and to be alive and free of cancer.  There is direct evidence that cases and controls were similar, which is they were recruited from the same eligible population, same area, and similar age. |
| **Confounding bias**  Did the study design or analysis account for important confounding and modifying variables? | Probably high risk of bias | The study was adjusted by age stratification.  Other confounding factors weren’t adjusted in the model. |
| **Attrition/ Exclusion bias**  Were outcome data complete without attrition or exclusion from analysis? | Probably high risk of bias | Not specified. |
| **Detection bias**  1. Can we be confident in the exposure characterization? | Definitely low risk of bias | Exposure is defined as the duration of work. The work history was collected according to the type of ship. Information was collected from the files of the Seamen’s Pension Fund and the National Board of Navigation.  There is a direct method to indicate exposure and there is no gold standard to measure the exposure. |
| 2. Can we be confident in the outcome assessment? | Definitely low risk of bias | Pancreatic cancer is defined as any case with code 157 of the International Classification of Diseases (9^th^ revision).  The definition is well-documented. |
| **Selective reporting bias**  Were all measured outcomes reported? | Definitely low risk of bias | The outcome was reported as odds ratio and 95% confidence interval.  This outcome provides sufficient detail to be included in the meta-analysis. |
| **Other bias**  Conflict of interest | Probably high risk of bias | Not specified. |

**Table S4Y.** Risk of bias of Sauni et al., 2017, according instructions report based on the OHAT risk of bias tool.

| **Bias domain** | **Risk of Bias** | **Comments** |
| --- | --- | --- |
| **Selection bias**  Did selection of study participants result in appropriate comparison groups? | Definitely low risk of bias | The participants were males employed for at least one year at the Kokkola cobalt plant (Freeport Cobalt Oy) during the period 1968–2004.  There is direct evidence that exposed and unexposed were similar, which is they were recruited from the same eligible population, same area, and similar age. |
| **Confounding bias**  Did the study design or analysis account for important confounding and modifying variables? | Probably high risk of bias | The study was adjusted by age stratification.  Other confounding factors weren’t adjusted in the model. |
| **Attrition/ Exclusion bias**  Were outcome data complete without attrition or exclusion from analysis? | Probably high risk of bias | Not specified. |
| **Detection bias**  1. Can we be confident in the exposure characterization? | Definitely low risk of bias | This study categorized exposure according to the department in which they had started working during their employment at the plant. Exposure in different departments was classified according to industrial hygienic measurements and biological monitoring.  There is a direct method to indicate exposure and there is no gold standard to measure the exposure. |
| 2. Can we be confident in the outcome assessment? | Probably low risk of bias | Pancreatic cancer death was assessed by the death certificate. |
| **Selective reporting bias**  Were all measured outcomes reported? | Definitely low risk of bias | The outcome was reported as standardized incidence ratio and 95% confidence interval.  This outcome provides sufficient detail to be included in the meta-analysis. |
| **Other bias**  Conflict of interest | Definitely high risk of bias | The author declared that two of his co-authors had been employed by Boliden. Boliden and the Kokkola Cobalt Plant are located in the same Kokkola Industrial Park area. Boliden has provided occupational health services to the Kokkola Cobalt Plant until the end of 2016. |

**Table S4Z.** Risk of bias of Selenskas et al., 1995, according instructions report based on the OHAT risk of bias tool.

| **Bias domain** | **Risk of Bias** | **Comments** |
| --- | --- | --- |
| **Selection bias**  Did selection of study participants result in appropriate comparison groups? | Definitely low risk of bias | The participants were employees from a cohort who had worked for 7 months or more at the Bound Brook plant between 1946 and 1967. Cases are defined as men who died with pancreatic cancer and had at least one hourly job assignment during that time period. Five controls per case were randomly selected from members of the cohort study with the same year of birth and who had survived to the age of at least as long as the cases.  There is direct evidence that cases and controls were similar, which is they were recruited from the same eligible population, same area, and similar age. |
| **Confounding bias**  Did the study design or analysis account for important confounding and modifying variables? | Probably low risk of bias | This study matched cases and controls with the same year of birth and age.  There is no adjustment for other confounding factors. |
| **Attrition/ Exclusion bias**  Were outcome data complete without attrition or exclusion from analysis? | Probably high risk of bias | Not specified. |
| **Detection bias**  1. Can we be confident in the exposure characterization? | Definitely low risk of bias | The exposure assessment for this study was conducted by the characterization of the workplace. Study participants were initially classified into major production and nonproduction work areas based on their having ever been assigned to these areas. The major production work process areas included: resins and varnish, vinyl and polyethylene processing, phenol, formaldehyde, hexamethylenetetramine, resin pulverizing, polystyrene, phenoxy resin, and fibers and fabrics. Nonproduction work areas were engineering, maintenance, offices, distribution, plant service, and research and development.  There is a direct method to indicate exposure and there is no gold standard to measure the exposure. |
| 2. Can we be confident in the outcome assessment? | Probably low risk of bias | Pancreatic cancer death was identified by the death certificate.  There is no gold standard to assess the outcome. |
| **Selective reporting bias**  Were all measured outcomes reported? | Definitely low risk of bias | The outcome was reported as relative risk and 95% confidence interval.  This outcome provides sufficient detail to be included in the meta-analysis. |
| **Other bias**  Conflict of interest | Probably high risk of bias | Not specified. |

**Table S4Aa. Risk of bias of Silverstein et al., 1988, according instructions report based on the OHAT risk of bias tool.**

| **Bias domain** | **Risk of Bias** | **Comments** |
| --- | --- | --- |
| **Selection bias**  Did selection of study participants result in appropriate comparison groups? | Definitely low risk of bias | The participants were employees of union members whose year of termination from the plant was 1950 or later, who died between January 1, 1950 and June 30, 1982, and whose employment duration was at least 5 years.  There is direct evidence that exposed and unexposed were similar, which is they were recruited from the same eligible population, same area, and similar age. |
| **Confounding bias**  Did the study design or analysis account for important confounding and modifying variables? | Probably high risk of bias | The study was adjusted by age stratification.  Other confounding factors weren’t adjusted in the model. |
| **Attrition/ Exclusion bias**  Were outcome data complete without attrition or exclusion from analysis? | Probably high risk of bias | Not specified. |
| **Detection bias**  1. Can we be confident in the exposure characterization? | Definitely low risk of bias | Exposure assessment for this study is classified by jobs using straight oil cutting fluids and jobs predominantly using water-based cutting fluids. Exposure group defined as employees who had cumulative weighted exposure duration exceeded 5 or 10 years. The non-exposure group defined as employees who had cumulative weighted duration in the exposure of interest and in others known or suspected of association with the cause of death under study was less than 12 months.  There is a direct method to indicate exposure and there is no gold standard to measure the exposure. |
| 2. Can we be confident in the outcome assessment? | Definitely low risk of bias | Pancreatic cancer is defined as any case with code 157 of the International Classification of Diseases (6^th^, 7^th^, or 8^th^ revision).  The definition is well-documented. |
| **Selective reporting bias**  Were all measured outcomes reported? | Definitely low risk of bias | The outcome was reported as odds ratio and 95% confidence interval.  This outcome provides sufficient detail to be included in the meta-analysis. |
| **Other bias**  Conflict of interest | Probably high risk of bias | Not specified. |

**Table S4Ab.** Risk of bias of Steenland et al., 2012, according instructions report based on the OHAT risk of bias tool.

| **Bias domain** | **Risk of Bias** | **Comments** |
| --- | --- | --- |
| **Selection bias**  Did selection of study participants result in appropriate comparison groups? | Definitely low risk of bias | The participants were DuPont employees who had worked at least 1 day at the plant between 1948 and 2002.  There is direct evidence that exposed and unexposed were similar, which is they were recruited from the same eligible population, same area, and similar age. |
| **Confounding bias**  Did the study design or analysis account for important confounding and modifying variables? | Probably high risk of bias | The study was adjusted by age stratification.  Other confounding factors weren’t adjusted in the model. |
| **Attrition/ Exclusion bias**  Were outcome data complete without attrition or exclusion from analysis? | Definitely low risk of bias | A worker with less than 50% of their work time in known jobs and departments and a missing date of birth was excluded from the analysis. |
| **Detection bias**  1. Can we be confident in the exposure characterization? | Definitely low risk of bias | Exposure assessments were conducted via life-table analyses, using cumulative serum levels derived from the job exposure matrix.    There is a direct method to indicate exposure. |
| 2. Can we be confident in the outcome assessment? | Definitely low risk of bias | Pancreatic cancer is defined as any case with code 157 of the International Classification of Diseases (9^th^ revision).  The definition is well-documented. |
| **Selective reporting bias**  Were all measured outcomes reported? | Definitely low risk of bias | The outcome was reported as standardized mortality ratio and 95% confidence interval.  This outcome provides sufficient detail to be included in the meta-analysis. |
| **Other bias**  Conflict of interest | Probably high risk of bias | Not specified. |

**Table S4Ac.** Risk of bias of Teta et al., 1993, according instructions report based on the OHAT risk of bias tool.

| **Bias domain** | **Risk of Bias** | **Comments** |
| --- | --- | --- |
| **Selection bias**  Did selection of study participants result in appropriate comparison groups? | Definitely low risk of bias | The participants were recruited from the company records and the National Death Index from 1 January 1979 to 31 December 1988. The study population worked in chemical manufacturing. Exposure is defined as a worker who has been assigned to an ethylene oxide unit. Non-exposure is defined as a worker who has not been assigned to an ethylene oxide unit.  There is direct evidence that exposed and unexposed were similar, which is they were recruited from the same eligible population, same area, and similar age. |
| **Confounding bias**  Did the study design or analysis account for important confounding and modifying variables? | Probably low risk of bias | This study was adjusted for age, calendar period, and interval since assignment.  This study considers only a partial list of covariates in the final analysis. |
| **Attrition/ Exclusion bias**  Were outcome data complete without attrition or exclusion from analysis? | Definitely low risk of bias | A worker with assignments in the chlorohydrin unit was excluded to avoid confounding. |
| **Detection bias**  1. Can we be confident in the exposure characterization? | Definitely low risk of bias | Exposure assessment was a monitoring program using an ethylene oxide unit. The estimated exposure ranges for units producing or using ethylene oxide during the period covered by this study are: 1925–1939, >14 ppm; 1940–1956, 14 ppm; 1957–1973, 5–10 ppm; and 1974–1988, <1 ppm, with frequent peaks of several hundred ppm in the earliest period and some peaks of similar intensity in the 1940s to mid-1950s.    There is a direct method to indicate exposure. |
| 2. Can we be confident in the outcome assessment? | Probably low risk of bias | Pancreatic cancer death was indicated by death certificate.  There is no gold standard for indicating outcome. |
| **Selective reporting bias**  Were all measured outcomes reported? | Definitely low risk of bias | The outcome was reported as relative risk and 95% confidence interval.  This outcome provides sufficient detail to be included in the meta-analysis. |
| **Other bias**  Conflict of interest | Probably high risk of bias | Not specified. |

**Table S4Ad.** Risk of bias of Van Barneveld et al., 2004, according instructions report based on the OHAT risk of bias tool.

| **Bias domain** | **Risk of Bias** | **Comments** |
| --- | --- | --- |
| **Selection bias**  Did selection of study participants result in appropriate comparison groups? | Definitely low risk of bias | The participantts were recruited from two public research institutes (the Netherlands Cancer Institute and the National Institute of Public Health and the Environment) and two universities (Leiden University and the Agricultural University Wageningen) that met the inclusion criteria of institutes that had traditionally carried out biological, biomedical, biochemical, or agronomic research as their main activity and had been in existence for at least 10 years. Exposure group defined as laboratory research. Non-exposure group defined as non-laboratory research division or faculties.  There is direct evidence that exposed and unexposed were similar, which is they were recruited from the same eligible population, same area, and similar age. |
| **Confounding bias**  Did the study design or analysis account for important confounding and modifying variables? | Probably low risk of bias | This study was adjusted for potential confounders based on a significance level set to 0.20, including age. |
| **Attrition/ Exclusion bias**  Were outcome data complete without attrition or exclusion from analysis? | Definitely low risk of bias | One institute was excluded because the institute was closed. Visiting scientists, trainees, and students were excluded. |
| **Detection bias**  1. Can we be confident in the exposure characterization? | Definitely low risk of bias | Exposure assessments were classified by faculty. Employees who had worked at biological, biomedical, biochemical, or agronomic research units were classified as exposure groups. Employees who had worked in the divisions of mathematics, computer sciences, epidemiology, psychosocial oncology, and desk research in the fields of health policy and environmental sciences were classified as non-exposure groups.    There is a direct method to indicate exposure. |
| 2. Can we be confident in the outcome assessment? | Definitely low risk of bias | Pancreatic cancer is defined as any case with code 157 of the International Classification of Diseases (9^th^ revision).  The definition is well-documented. |
| **Selective reporting bias**  Were all measured outcomes reported? | Definitely low risk of bias | The outcome was reported as relative risk and 95% confidence interval.  This outcome provides sufficient detail to be included in the meta-analysis. |
| **Other bias**  Conflict of interest | Probably high risk of bias | Not specified. |

**Table S4Ae.** Risk of bias of Zhang et al., 2005, according instructions report based on the OHAT risk of bias tool.

| **Bias domain** | **Risk of Bias** | **Comments** |
| --- | --- | --- |
| **Selection bias**  Did selection of study participants result in appropriate comparison groups? | Probably low risk of bias | Cases were identified through the State Health Registry of Iowa and diagnosed between August 1985 and December 1987. All cases who were histologically confirmed as having pancreatic adenocarcinoma were aged between 40 and 85 years old and were residents of Iowa. Controls were matched by gender and 5-year age group to all cases in the larger study. Controls were selected randomly from computerized state driver’s license records for persons younger than age 65 and from the United State Centers for Medicare and Medicaid Service.  There is indirect evidence that cases and controls were similar, which is they were recruited from the same age. |
| **Confounding bias**  Did the study design or analysis account for important confounding and modifying variables? | Definitely low risk of bias | This study matched age and sex with cases and controls. The potential confounder was adjusted for age, red meat intake, fruit intake, leisure time physical activity, having a first-degree relative with pancreatic cancer, and tobacco smoking. |
| **Attrition/ Exclusion bias**  Were outcome data complete without attrition or exclusion from analysis? | Definitely low risk of bias | Control participantts who had previous cancer diagnoses other than nonmelanoma skin cancer were excluded. |
| **Detection bias**  1. Can we be confident in the exposure characterization? | Definitely low risk of bias | Exposure assessment was indicated by industry and job title. Industries and job titles were coded according to schemes in the 1987 edition of the Standard Industry Classification and the 1980 Standard Occupational Classification Manual. The duration of employment (<10 years and ≥10 years) was used to evaluate the risk of pancreatic cancer.    There is a direct method to indicate exposure. |
| 2. Can we be confident in the outcome assessment? | Probably low risk of bias | Outcome was assessed by histologically confirmed as having pancreatic adenocarcinoma.  There is no gold standard to assess the outcome. |
| **Selective reporting bias**  Were all measured outcomes reported? | Definitely low risk of bias | The outcome was reported as odds ratio and 95% confidence interval.  This outcome provides sufficient detail to be included in the meta-analysis. |
| **Other bias**  Conflict of interest | Probably high risk of bias | Not specified. |

# **Section 5: Certainty of evidence**

The Grading of Recommendations, Assessment, Development and Evaluations (GRADE) rating (4) reflects the overall certainty of evidence of the final findings that show the true association between the exposure and the outcome, which is rated as “high,” “moderate,” “low,” or “very low.” Observational studies received an initial rating of “moderate.” The rating can be decreased if there are concerns about the risk of bias, inconsistency, indirectness, imprecision, or publication bias. The rating can be increased when there are large effects, dose-response relationships, and plausible confounders.

A summary of findings regarding the certainty of evidence is provided in Table S5. Regarding the risk-of-bias rating for studies investigating pancreatic cancer risk, since most studies were categorized as Tier 1 or Tier 2 risk of bias, the overall risk of bias was “serious,” indicating plausible bias that raises some doubt about the results. The inconsistency of certainty assessment indicated moderate heterogeneity in meta-analyses of pancreatic cancer (*I^2^* = 51%). However, the explanation for this heterogeneity could reveal some inconsistencies, such as differences in exposure assessments, differences in exposure intensities, and differences in the types of industries and chemical agents. The use of different exposure assessments may introduce misclassification bias of exposure. Other reasons may not to be a source of heterogeneity due to their results in sub-group analyses are consistent with pooled relative risk. The respective category of inconsistency of certainty assessment was “not serious.” The indirectness and imprecision certainty assessment indicated a category of “not serious” because the outcome (pancreatic cancer) of our study showed the evidence answers directly to workers exposed to chemical agents, and the confidence interval of pooled analysis is narrow (1.05–1.10). Funnel plots exhibited symmetrical patterns, and Begg’s tests yielded no evidence of publication bias, indicating an undetectable publication bias. We observed the dose-response gradient association between occupational exposure duration to chemical agents and pancreatic cancer and the residual toward the null, suggesting an increase in the certainty of evidence rating.

In summary, the risk of bias made our rating for the certainty of evidence from moderate to low certainty of evidence for the association between occupational exposure duration to chemical agents and pancreatic cancer. However, the observation of a dose-response gradient association between occupational exposure duration to chemical agents and pancreatic cancer and residual toward the null made our rating for the certainty of evidence from low to moderate for the associations. Thus, the final rating of the certainly of evidence was moderate for the association between occupational exposure duration to chemical agents and pancreatic cancer.

**Table S5.** Summary of the certainty of evidence.

| **Certainty assessment** | | | | | | | **No. of participants** | | **Effect** | **Certainty of evidence (GRADE)** | |
| --- | --- | --- | --- | --- | --- | --- | --- | --- | --- | --- | --- |
| **Number of studies** | **Study design** | **Risk of bias** | **Inconsistency** | **Indirectness** | **Imprecision** | **Other considerations** | **Workers who had exposed to chemical** | **Workers who had not exposed to chemical** | **Relative Risk  (95% CI)** |  | |
| Pancreatic cancer (follow-up period: from 1980 to 2020) | | | | | | | | | | |  |
| 31 | Observational studies | Serious | Not serious | Not serious | Not serious | Residual bias towards the null^a^  Dose response gradient | 140,665 | 165,213 | 1.08  (1.05–1.10) | ⨁⨁⨁◯ **Moderate**  Due to risk of bias | |

^a^ This study addressed the healthy worker effect; workers often exhibit lower overall death rates than the general population because workers may leave their jobs owing to perceived or actual health consequences. The population in this study are workers among both exposed and unexposed. Abbreviations: CI, confidence interval; GRADE, Grading of Recommendations Assessment, Development, and Evaluation.

# **Section 6: The flowchart for agreement during screening and selection of systematic review**

**
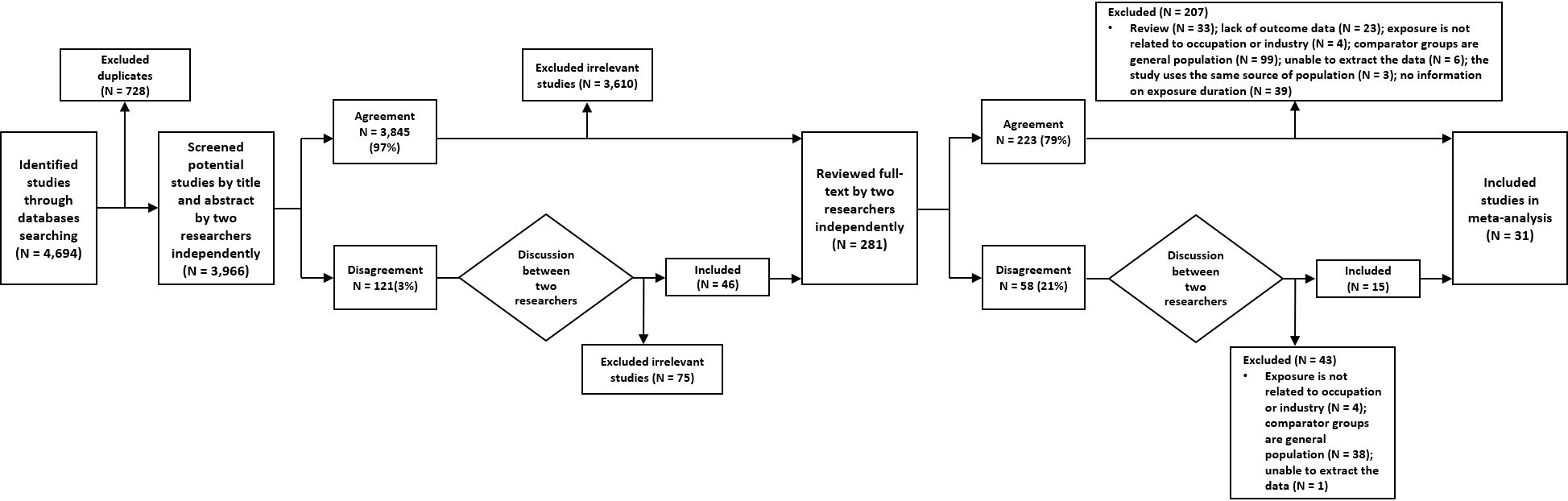
**

**Figure S1.** The summary of agreement during screening and selection process of systematic review.

Abbreviations: N, number of studies

# **Section 7: The characteristics of studies included in the meta-regression and meta-analysis**

**Table S6.** A summary of studies investigating the association between occupational exposure and pancreatic cancer incidence and mortality.

| **Authors** | **Location** | **Industry type/ occupations** | **Related-chemical agents** | **Participant enrollment, study types, effect measure types, and confounders** | **Population and number of participants** | **Exposure assessment, employment years, and outcome assessment** |
| --- | --- | --- | --- | --- | --- | --- |
| Alguacil J et al., 2000 | Eastern part of Spain | Occupational exposure in any of ten activities: pesticide use, handling of petroleum derivatives, chemical industry, metal industry, rubber industry, graphic arts, jewelry, manufacture or repair of auto-mobiles, leather tanning and textile industry | Not reported | **Participant enrollment:** 1992–1995  **Study type:** Case control  **Effect measure type:** Odds ratio  **Confounders:** Age, hospital, smoking, coffee consumption, and alcohol use | **Population:** Males and females have a mean aged of 67 for cases and 61 for controls  **Number of participants:** 164 cases and 238 controls | **Exposure assessment:** Interview by using questionnaire and classify based on job-title  **Employment years:** At least 6 months (164 cases and 238 controls) and at least 10 years (164 cases and 238 controls)  **Outcome assessment:** Diagnosed by physician |
| Alguacil et al., 2000 | Eastern part of Spain | Occupational exposure in any of ten activities: pesticide use, handling of petroleum derivatives, chemical industry, metal industry, rubber industry, graphic arts, jewelry, manufacture or repair of auto-mobiles, leather tanning and textile industry | Pesticide, hydrocarbon solvents, aluminum, chromium, lead, asbestos, PAHs, cotton dust, and cutting oils | **Participant enrollment:** 1992–1995  **Study type:** Case control  **Effect measure type:** Odds ratio  **Confounders:** Age, hospital, smoking, coffee consumption, and alcohol use | **Population:** Males and females have a mean aged of 67 for cases and 61 for controls  **Number of participants:** 164 cases and 238 controls | **Exposure assessment:** The Finnish Job-Exposure Matrix  **Employment years:** < 20 years and ≥ 20 years; number of subjects not reported  **Outcome assessment:** A panel of experts reviewed all clinical and pathological data available |
| Bardin et al., 1997 | Michigan, the United States | Automobile industry | Metal | **Participant enrollment**: 1917–1984  **Study type:** Case control  **Effect measure type:** Odds ratio  **Confounders:** Race, sex, plant, and date of birth (±5 years) | **Population:** Males and females whose ages are not specified  **Number of participants:** 97 cases and 1,825 controls | **Exposure assessment:** Job-exposure matrix  **Employment years:** 0 to < 10 years and 10 to 20 years; number of subjects not reported  **Outcome assessment:** Death certificate |
| Beane Freeman et al., 2011 | The United States | Agriculture | Pesticide (atrazine) | **Participant enrollment**: 1994–2007  **Study type:** Cohort  **Effect measure type:** Relative risk  **Confounders:** Race, sex, age, smoking history, alcohol use, education, state of residence, family history of cancer, applicator type, and ever use of other pesticides | **Population:** Males and females whose ages are not specified  **Number of participants:** 53,662 | **Exposure assessment:**  Self-administered questionnaire  **Employment years:** less than one year; 51 cases  **Outcome assessment:** Death certificate |
| Beard et al., 2003 | Australia | Agriculture | Pesticides (DDT) | **Participant enrollment**: 1935–1995  **Study type:** Case control  **Effect measure type:** Relative risk  **Confounders:** Age, sex, and smoking | **Population:** Males whose ages are not specified  **Number of participants:** 144 | **Exposure assessment:**  Questionnaire  **Employment years:**  < 5 to < 15 years; number of subjects not reported  **Outcome assessment:** Cancer registry |
| Benson et al., 1993 | The United States | Chemical industry | Ethylene oxide | **Participant enrollment**: 1940–1967  **Study type:** Cohort  **Effect measure type:** Relative risk  **Confounders:** Age, sex, calendar period, and interval since assignments | **Population:** Males aged between 23 and 83 years  **Number of participants:** 278 | **Exposure assessment:** Job assignment in the chlorohydrin unit  **Employment year:** 0 to < 10 years and 10 to > 20 years; number of subjects not reported  **Outcome assessment:** Death certificate |
| De Roos et al., 2000 | Iowa and North Carolina, the United States | Agriculture | Pesticides | **Participant enrollment**: 1993–1997  **Study type:** Cohort  **Effect measure type:** Relative risk  **Confounders:** Age at enrollment, education, pack-years of cigarette smoking, alcohol consumption in the past year, family history of cancer in first-degree relatives, state of residence, and five pesticides | **Population:** Males and females aged of 40–70 years  **Number of participants:** 53,656 | **Exposure assessment:** Questionnaire  **Employment year:** 0 to 10 years (25 cases)  **Outcome assessment:** ICD–9, coded 157 |
| Garabrant et al., 1992 | The United States | Chemical industry | Pesticides (DDT) | **Participant enrollment**: 1948–1971  **Study type:** Case control  **Effect measure type:** Relative risk  **Confounders:** Age and sex | **Population:** Males have a mean age of 60.4 years  **Number of participants:** 28 cases and 112 controls | **Exposure assessment:** Questionnaire  **Employment years:** 0 to 10 years (11 cases and 20 controls)  **Outcome assessment:** Histologically confirmed |
| Greenberg et al., 2000 | West Virginia, the United States | Chemical manufacturing | Ethylene oxide | **Participant enrollment**: 1940–1978  **Study type:** Cohort  **Effect measure type:** Relative risk  **Confounders:** Age, sex, calendar period, and interval since hire | **Population:** Males whose ages are not specified  **Number of participants:** 2,174 | **Exposure assessment:** Questionnaire  **Employment years:** 0 to < 10 years and 10 to > 20 years; number of subjects not reported  **Outcome assessment:** Death certificate |
| Hidajat et al., 2019 | United Kingdom | Rubber factory | Rubber dust and fumes | **Participant enrollment**: 1967–2015  **Study type:** Cohort  **Effect measure type:** Hazard ratio  **Confounders:** Age and birth year | **Population:** Males and females aged 35 years or older  **Number of participants:** 36,443 | **Exposure assessment:** Job-exposure matrix  **Employment years:** 9 to > 27 years (328 subjects)  **Outcome assessment:** Cancer registry |
| Kauppien, 1995 | Finland | Any type of industries or occupations | Inorganic dust containing crystalline silica, lead and lead compounds, pesticides, and solvent | **Participant enrollment**: 1984–1987  **Study type:** Case control  **Effect measure type:** Odds ratio  **Confounders:** Age, sex, smoking in the 1960s, history of diabetes mellitus, and alcohol consumption in the 1960s. | **Population:** Males and females aged 40–74 years  **Number of participants:** 1,419 cases and 1,622 controls | **Exposure assessment:** Job-exposure matrix  **Employment year:** At least 10 years (165 cases)  **Outcome assessment:** ICD–9, coded 157 |
| Kolstad et al., 1995 | Denmark | Reinforced plastic industry | Styrene | **Participant enrollment**: 1970–1989  **Study type:** Cohort  **Effect measure type:** Relative risk  **Confounders:** Age | **Population:** Males whose ages are not specified  **Number of participants:** 1,663 | **Exposure assessment:** Job-exposure matrix  **Employment years:** < 1 and ≥ 1 years; number of subjects not reported  **Outcome assessment:** ICD–7, coded 157 |
| Lerro et al., 2020 | The United States | Agriculture | Herbicide | **Participant enrollment**: 1993–1997  **Study type:** Cohort  **Effect measure type:** Relative risk  **Confounders:** Age, sex, race, state, applicator type, education, imazethapyr, smoking, and family history of cancer. | **Population:** Males and females whose ages are not specified  **Number of participants:** 49,922 | **Exposure assessment:** Computer assisted telephone interviews  **Employment years:** < 1 and > 10 years (86 non-exposure and 77 exposure groups)  **Outcome assessment:** ICD–O–3 |
| Li et al., 2006 | China | Textile industry | Not reported | **Participant enrollment**: 1989–1991  **Study type:** Case control  **Effect measure type:** Odds ratio  **Confounders:** Age, sex, and smoking | **Population:** Females whose ages are not specified  **Number of participants:** 180 cases and 3,183 controls | **Exposure assessment:** Employment records and interviews  **Employment years:** < 10 (41 cases and 824 controls), 10–20 (59 cases and 1,055 controls), and > 20 years (122 cases and 2,043 controls)  **Outcome assessment:** ICD–9, coded 157 |
| Loomis et al., 2019 | Denmark, Finland, Italy, Norway, Sweden, the United Kingdom | Reinforced plastic industry | Styrene | **Participant enrollment**: 1945–1991  **Study type:** Cohort  **Effect measure type:** Relative risk  **Confounders:** Age | **Population:** Males and females whose ages are not specified  **Number of participants:** 37,021 | **Exposure assessment:** Job-exposure matrix  **Employment years:** 2–5 (8 subjects), 0 to < 10, and 10 to > 20 years; number of subjects not reported for < 10 and > 20 years  **Outcome assessment:** ICD–8, coded 157 |
| Lundin et al., 2009 | Minnesota, the United States | 3M Company manufacturing facility | Ammonium perfluorooctanoate | **Participant enrollment**: 1997–2002  **Study type:** Cohort  **Effect measure type:** Hazard ratio  **Confounders:** Age, sex, and year of birth | **Population:** Males and females whose ages are not specified  **Number of participants:** 3,993 | **Exposure assessment:** Job-exposure matrix  **Employment years:** < 1 (7 cases) and > 5 years (6 cases)  **Outcome assessment:** ICD |
| Lynge et al., 2006 | Nordic country | Dry cleaning | Not reported | **Participant enrollment**: 1970–2000  **Study type:** Case control  **Effect measure type:** Relative risk  **Confounders:** Age, sex, smoking status, and alcohol consumption | **Population:** Males and females whose ages are not specified  **Number of participants:** 229 cases and 891 controls | **Exposure assessment:** Blinded personal telephone interview  **Employment years:** 0–1 (6 cases and 12 controls) and ≥ 10 years (51 cases and 172 controls)  **Outcome assessment:** ICD |
| Marsh et al., 2007 | The United States | The wastewater, pulp and paper, textile, chemical,  mining, and oil industries. | Acrylamide | **Participant enrollment**: 1925–2002  **Study type:** Cohort  **Effect measure type:** Relative risk  **Confounders:** Age and year of birth | **Population:** Males whose ages are not specified  **Number of participants:** 8,852 | **Exposure assessment:** Job-exposure matrix  **Employment years:** <1 (22 cases) and 1 to >15 years (32 cases)  **Outcome assessment:** ICD |
| Mikoczy et al., 1996 | Sweden | Leather tanning industry | Aniline and  azo dyes, Chrome salts, Formaldehyde, Chlorophenols, Mercury, Organic solvents, Arsenic, Wood dust, and Leather dust | **Participant enrollment**: 1900–1989  **Study type:** Case control  **Effect measure type:** Odds ratio  **Confounders:** Age, sex, and plant | **Population:** Males and females whose ages are not specified  **Number of participants:** 68 cases and 178 controls | **Exposure assessment:** Job-exposure matrix  **Employment years:** At least one year (14 cases and 30 controls)  **Outcome assessment:** ICD–7 |
| Olsen et al., 1997 | The United States | Not specified | Ethylene and propylene chlorohydrin production | **Participant enrollment**: 1940–1992  **Study type:** Cohort  **Effect measure type:** Relative risk  **Confounders:** Age, sex, calendar period, manufacturing location, and period of employment. | **Population:** Males whose ages are not specified  **Number of participants:** 1,361 | **Exposure assessment:** Job-exposure matrix  **Employment years:** 10 to 20 years; number of subjects not reported  **Outcome assessment:** ICD–8 |
| Reul et al., 2016 | Shanghai, China | Textile | Not reported | **Participant enrollment**: 1989–1991  **Study type:** Case-cohort  **Effect measure type:** Hazard ratio  **Confounders:** Age and smoking | **Population:** Females whose ages are not specified  **Number of participants:** 481 cases and 3,179 controls | **Exposure assessment:**  Job-exposure matrix  **Employment years:** 0–10 (67 cases and 639 controls), 10–20 (101 cases and 762 controls), and > 20 years (259 cases and 1,450 controls)  **Outcome assessment:** Manual review of medical records |
| Romundstad et al., 2000 | Norway | Aluminum plants | PAHs | **Participant enrollment**: 1953–1996  **Study type:** Cohort  **Effect measure type:** Relative risk  **Confounders:** Age and smoking | **Population:** Males whose ages are not specified  **Number of participants:** 11,103 | **Exposure assessment:**  Job-exposure matrix  **Employment years:** 10 to < 20 and 20 to 30 years; number of subjects not reported  **Outcome assessment:** ICD–7 |
| Romundstad et al., 2000 | Norway | Aluminum reduction plant | PAHs | **Participant enrollment**: 1962–1995  **Study type:** Cohort  **Effect measure type:** Relative risk  **Confounders:** Age and smoking | **Population:** Males whose ages are not specified  **Number of participants:** 5,627 | **Exposure assessment:**  Job-exposure matrix  **Employment years:** 0 to 10 years; number of subjects not reported  **Outcome assessment:** ICD–7 |
| Saarni et al., 2002 | Finland | Seafarer | Not reported | **Participant enrollment**: 1960–1980  **Study type:** Cohort  **Effect measure type:** Odds ratio  **Confounders:** Age | **Population:** Males whose ages not specified  **Number of participants:** 232 | **Exposure assessment:**  Job-exposure matrix  **Employment years:** ≥ 1 month and ≥ 3 years; number of subjects not reported  **Outcome assessment:** ICD–9 code 157 |
| Sauni et al., 2017 | Finland | Metal industry | Cobalt metal | **Participant enrollment**: 1969–2013  **Study type:** Cohort  **Effect measure type:** Relative risk  **Confounders:** Age | **Population:** Males whose ages not specified  **Number of participants:** 995 | **Exposure assessment:**  Job-exposure matrix  **Employment years:** > 1 (69 cases and 217 controls) and > 3 (39 cases and 139 controls) years  **Outcome assessment:** Finnish Cancer Registry |
| Selenskas et al., 1995 | New Jersey, the United States | Processing resin synthetic industry | Vinyl and polyethylene, resin, and varnish | **Participant enrollment**: 1946–1988  **Study type:** Cohort  **Effect measure type:** Relative risk  **Confounders:** Matched with same year of birth and age | **Population:** Males and females whose ages not specified  **Number of participants:** 168 | **Exposure assessment:**  Job-exposure matrix  **Employment years:** ≤ 1 year and > 16 years; number of subjects not reported  **Outcome assessment:** Death certificate |
| Silverstein et al., 1988 | The United States | Bearing plant | Metalworking fluids and abrasive | **Participant enrollment**: 1950–1982  **Study type:** Cohort  **Effect measure type:** Odds ratio  **Confounders:** Age | **Population:** Males and females whose ages not specified  **Number of participants:** 1,766 | **Exposure assessment:**  Job-exposure matrix  **Employment years:** ≤ 10 year and > 10 years; number of subjects not reported  **Outcome assessment:** ICD–7–8 |
| Steenland et al., 2012 | West Virginia, the United States | Chemical plants | Perfluorooctanoic Acid | **Participant enrollment**: 1952–2008  **Study type:** Cohort  **Effect measure type:** Relative risk  **Confounders:** Age | **Population:** Males and females whose ages not specified  **Number of participants:** 5,791 | **Exposure assessment:**  Job-exposure matrix  **Employment years:** Less than one year (18 cases)  **Outcome assessment:** ICD–9 |
| Teta et al., 1993 | The United States | Chemical manufacturing | Ethylene oxide | **Participant enrollment**: 1979–1988  **Study type:** Cohort  **Effect measure type:** Relative risk  **Confounders:** Age, sex, calendar period, and interval since assignment | **Population:** Males and females whose ages not specified  **Number of participants:** 440 | **Exposure assessment:**  Job-exposure matrix  **Employment years:** 0 to < 10 years and 10 to 20 years; number of subjects not reported  **Outcome assessment:** Death certificates |
| Van Barneveld et al., 2004 | Netherlands | Biology research laboratory | Not reported | **Participant enrollment**: 1960–1992  **Study type:** Cohort  **Effect measure type:** Odds ratio  **Confounders:** Age | **Population:** Males and females whose ages not specified  **Number of participants:** 9,711 | **Exposure assessment:**  Job-exposure matrix  **Employment years:** 10 to 20 years (5 cases and 5 controls)  **Outcome assessment:** ICD–9 |
| Zhang et al., 2005 | The United States | Any type of industries or occupations | Not reported | **Participant enrollment**: 1985–1987  **Study type:** Case control  **Effect measure type:** Relative risk  **Confounders:** Age, red meat intake, fruit intake, leisure time physical activity, having a first-degree relative with pancreatic cancer, and tobacco smoking | **Population:** Males whose ages more than 40 years  **Number of participants:** 376 cases and 2,434 controls | **Exposure assessment:**  Occupations were categorized based on the Standard Occupational Classification and Standard Industry Classification. Past occupational exposure was assessed by interview  **Employment years:** < 10 (10 cases and 25 controls) and ≥ 10 (12 cases and 53 controls) years  **Outcome assessment:** Histologically confirmed |

Abbreviations: DDT, dichloro-diphenyl-trichloroethane; ICD, International Classification of Diseases; PAHs, Polycyclic Aromatic Hydrocarbons

# **Section 8: The percentage of total weight across the 31 included studies**

**Table S7**. The summary of percentage of weight of each studies included in the meta-analysis

| **Authors** | **Country** | **Percentage of weight** |
| --- | --- | --- |
| Alguacil J et al., 2000 | Spain | 1.28 |
| Alguacil et al., 2000 | Spain | 0.29 |
| Bardin et al., 1997 | Michigan, the United States | 77.79 |
| Beane Freeman et al., 2011 | The United States | 0.62 |
| Beard et al., 2003 | Australia | 0.07 |
| Benson et al., 1993 | The United States | 0.04 |
| De Roos et al., 2000 | Iowa and North Carolina, the United States | 0.13 |
| Garabrant et al., 1992 | The United States | 0.07 |
| Greenberg et al., 2000 | West Virginia, the United States | 0.1 |
| Hidajat et al., 2019 | United Kingdom | 3.58 |
| Kauppien, 1995 | Finland | 1.5 |
| Kolstad et al., 1995 | Denmark | 0.18 |
| Lerro et al., 2020 | The United States | 0.74 |
| Li et al., 2006 | China | 2.29 |
| Loomis et al., 2019 | Denmark, Finland, Italy, Norway, Sweden, United Kingdom | 4.48 |
| Lundin et al., 2009 | Minnesota, the United States | 0.07 |
| Lynge et al., 2006 | Nordic country | 0.52 |
| Marsh et al., 2007 | The United States | 0.28 |
| Mikoczy et al., 1996 | Sweden | 0.08 |
| Olsen et al., 1997 | The United States | 0.02 |
| Reul et al., 2016 | Shanghai, China | 3.47 |
| Romundstad et al., 2000 | Norway | 0.28 |
| Romundstad et al., 2000 | Norway | 0.02 |
| Saarni et al., 2002 | Finland | 0.98 |
| Sauni et al., 2017 | Finland | 0.06 |
| Selenskas et al., 1995 | New Jersey, the United States | 0.24 |
| Silverstein et al., 1988 | The United States | 0.19 |
| Steenland et al., 2012 | West Virginia, the United States | 0.29 |
| Teta et al., 1993 | The United States | 0.07 |
| Van Barneveld et al., 2004 | Netherlands | 0.02 |
| Zhang et al., 2005 | The United States | 0.25 |

# **Section 9: Duration-response association between occupational exposure to chemical agent and pancreatic cancer risk by weighted random-effect dose response model**

A meta-regression across 15 studies was conducted to assess the dose-response association between exposure duration to chemical agents and pancreatic cancer risk in workers. We observed a positive dose-response association between exposure duration to chemical agents and the risk of pancreatic cancer in workers, with a 2% increase in the pooled RR (slope = 1.02; 95% CI = 1.01–1.04) per year. The meta-analysis revealed a significant association between exposure duration to chemical agents and pancreatic cancer risk among workers (pooled RR = 1.01; 95% CI = 1.00–1.02; *I^2^* = 0% and *τ^2^* = 0.0000).

We applied the sensitivity analysis to explore the robustness of the pooled RR by excluding the study that provided the largest weight to overall pooled RR. Overall, the pooled RRs of pancreatic cancer for exposure duration to chemical agents in workers were 1.01 (95% CI = 1.00–1.02; *I^2^* = 0%) across 15 studies in the main meta-analysis model. Among the included studies, one study contributed with the largest percentage to the total weight. By excluding this study from the alternative meta-analysis model for occupational exposure duration to chemical agents, the pooled RRs of pancreatic cancer differed from the main model by approximately 0%, with an RR of 1.01 (95% CI = 1.00–1.02; *I^2^* = 0%). The funnel plot for the meta-analysis showed symmetry for the effect estimates, and the Begg’s test suggested publication bias (P = 0.010).

**References**

1. Bonita R, Beaglehole R, Kjellström T. Basic epidemiology Geneva: World Health Organization; 2006.

2. Jones ME, Swerdlow AJ. Bias in the standardized mortality ratio when using general population rates to estimate expected number of deaths. Am J Epidemiol. 1998;148(10):1012-7.

3. OHAT. Handbook for Conducting a Literature-Based Health Assessment Using OHAT Approach for Systematic Review and Evidence Integration United States: National Toxicology Program U.S Department of Health and Human Services; 2019.

4. Schünemann HJ V GE, Glasziou, P., Akl, E.A., Skoetz, N., Guyatt, G.H. . Chapter 14: Completing ‘Summary of findings’ tables and grading the certainty of the evidence In: Higgins JPT, Thomas J, Chandler J, Cumpston M, Li T, Page MJ, Welch VA, editors. Cochrane Handbook for Systematic Reviews of Interventions version 6.1 (updated September 2020) Chichester (UK): John Wiley & Son; 2020.
